# Hierarchical joint analysis of marginal summary statistics—Part II: High‐dimensional instrumental analysis of omics data

**DOI:** 10.1002/gepi.22577

**Published:** 2024-06-17

**Authors:** Lai Jiang, Jiayi Shen, Burcu F. Darst, Christopher A. Haiman, Nicholas Mancuso, David V. Conti

**Affiliations:** ^1^ Department of Population and Public Health Sciences, Division of Biostatistics, Keck School of Medicine University of Southern California Los Angeles California USA; ^2^ Center for Genetic Epidemiology, Keck School of Medicine University of Southern California Los Angeles California USA; ^3^ Public Health Sciences Fred Hutchinson Cancer Center Seattle Washington USA; ^4^ Norris Comprehensive Cancer Center University of Southern California Los Angeles California USA

**Keywords:** hierarchical joint analysis of marginal summary data (hJAM), instrumental variable analysis, Mendelian randomization, omics data, summary statistics, transcriptome‐wide association study (TWAS)

## Abstract

Instrumental variable (IV) analysis has been widely applied in epidemiology to infer causal relationships using observational data. Genetic variants can also be viewed as valid IVs in Mendelian randomization and transcriptome‐wide association studies. However, most multivariate IV approaches cannot scale to high‐throughput experimental data. Here, we leverage the flexibility of our previous work, a hierarchical model that jointly analyzes marginal summary statistics (hJAM), to a scalable framework (SHA‐JAM) that can be applied to a large number of intermediates and a large number of correlated genetic variants—situations often encountered in modern experiments leveraging omic technologies. SHA‐JAM aims to estimate the conditional effect for high‐dimensional risk factors on an outcome by incorporating estimates from association analyses of single‐nucleotide polymorphism (SNP)‐intermediate or SNP‐gene expression as prior information in a hierarchical model. Results from extensive simulation studies demonstrate that SHA‐JAM yields a higher area under the receiver operating characteristics curve (AUC), a lower mean‐squared error of the estimates, and a much faster computation speed, compared to an existing approach for similar analyses. In two applied examples for prostate cancer, we investigated metabolite and transcriptome associations, respectively, using summary statistics from a GWAS for prostate cancer with more than 140,000 men and high dimensional publicly available summary data for metabolites and transcriptomes.

## 1 INTRODUCTION

**Figure 1:**
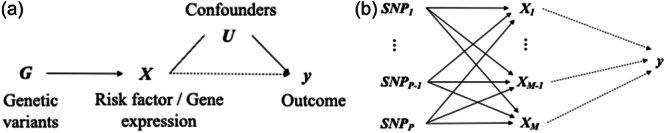
Direct acyclic graphs (DAG) for instrument variable analysis using genetic variants. (a) This DAG displays a general framework for Mendelian randomization (MR) and transcriptome‐wide association studies (TWAS). For MR and TWAS, *X* denotes a risk factor and a gene, respectively. *G* denotes the set of genetic variants that are included in the analysis, *U* denotes potential unobserved confounders, and *y* denotes the outcome. The solid and dotted lines define a strong or moderate causal effect and an uncertain association, respectively. (b) This DAG expands the framework in (a) into a high‐dimensional setting where a large number of intermediates and genetic variants (i.e., SNPs) exist. *X* can be genomics, transcriptomics, metabolomics, and so forth.

Instrumental variable (IV) analysis is a general approach to estimate causal relations in observational studies while controlling for unmeasured confounding, measurement error or reverse causality when required assumptions are met. Specifically, an IV is assumed not to have any direct effect on the outcome of interest. Instead, its effect on the outcome is entirely mediated through the dependent variables of interest, which are called intermediates (Chul, 2016; Mehta, 2001; Stock, 2001). IV analysis has been widely adopted not only in econometrics (Goldberger, 1972; Angrist, 2009) and health sciences (Newhouse & Mcclellan, 1998) but also in genetic studies where single‐nucleotide polymorphisms (SNPs) are used as IVs (Didelez & Sheehan, 2007; Greenland, 2000a). Both Mendelian randomization (MR) and transcriptome‐wide association studies (TWAS) can be considered as related applications of an IV analysis framework to obtain effect estimates of the intermediate–outcome relationship. While MR typically focuses on estimating the causal effect of modifiable risk factors on the outcome of interest, TWAS aims to discover genes regulated at the transcriptional level that are associated with complex traits and diseases. Thus, TWAS primarily conducts association tests, but MR aims to infer causal relationships. Though MR and TWAS are applied to address different research questions, they share the same IV analysis framework: risk factors in MR and gene expression levels in TWAS are both analogous to intermediates in IV analysis, as shown in Figure 1a. In the IV analysis framework, the causal estimand is the effect of intermediate(s) on the outcome. Similarly, in MR, the estimand is the effect size of the risk factors on the outcome, while in TWAS, it is the association between gene expression levels and the traits or diseases of interest.

For an IV analysis to be valid, the use of a genetic variant as a valid IV has to satisfy three conditions: (1) it can only be associated with the outcome through the intermediate (“exclusion restriction”); (2) there is a nonzero effect (i.e., association) between IV and the intermediate (“relevance”); and (3) the potential confounders between the intermediate and the outcome are independent of the genetic variable (“independence”) (Newhouse & McClellan, 1998). To avoid violating the first and third assumptions, researchers often focus on a single intermediate. As studies continue to obtain more genotype measurements to use as IV and more omic measurements to use as potential intermediates (e.g., metabolomics), there is the need for analytical methods that leverage genome‐wide data and account for multiple SNPs and multiple intermediates to provide unbiased estimates of effect.

Recent techniques have been developed to utilize multiple genetic variants (i.e., SNPs) instead of a single variant to provide more robust effect estimation (Burgess et al., 2013; Didelez & Sheehan, 2007; Lawlor et al., 2008; Smith, 2004). However, with the inclusion of more SNPs, these approaches are potentially more likely to violate the assumptions of MR. Horizontal pleiotropy is a common cause of false‐positive MR findings that occur when the IV impacts disease risk outside of the mediation pathway. Modern statistical techniques have been developed to address such bias and pleiotropy can potentially be identified by evaluating the impact of the invalid IVs. For example, Mendelian randomization with Egger (MR Egger) (Bowden et al., 2015) incorporates an intercept term to account for the pleiotropy; Mendelian randomization with pleiotropy residual sum and outlier detection (MR‐PRESSO) (Verbanck et al., 2018) removes detected outliers that may be due to pleiotropy and performs the analysis on the remaining SNPs as the instrument variables. Another way to account for pleiotropy is to include multiple correlated intermediates in the MR analysis (Figure 1b), such as hierarchical joint analysis of marginal summary statistics (hJAM) (Jiang et al., 2021) and multivariable inverse variance weighted Mendelian randomization (MVIVW) (Burgess & Thompson, 2015). However, while these approaches rely on linear regression, they are often limited to a few intermediates based on the stability of estimation with “*p* less than *n*” where “*p*” is the number of intermediates and “*n*” the number of informative SNPs as IVs. Thus, selection of both intermediates and SNPs leverage biology for the selection of both.

In recent years, high‐throughput technologies have been developed to perform large‐scale experiments measuring numerous omic types, including genomics (Bustamante et al., 2011), metabolomics (Dettmer et al., 2007), transcriptomics (Wang et al., 2009), proteomics (Tyers & Mann, 2003), and epigenomics (Sekula et al., 2016). Multi‐omics datasets are created by integrating multiple types of omic measurements from the same set of samples. Though collecting multi‐omic measurements from a large scale of participants can be expensive and time‐consuming, recent machine‐learning‐based approaches allow researchers to generate trait predictions from genotypes in large‐scale biobanks (Lambert et al., 2021; Mosley et al., 2018; Xu et al., 2023). Well‐validated predictions or imputations of multi‐omic traits from genetic data are of great interest to study the molecular mechanisms underly common diseases. Faced with such high‐dimensional data, approaches for analysis within an instrumental framework tend to treat each intermediate as independent and use an agnostic approach to select first for the relevant SNPs and then for the noteworthy intermediates. For example, commonly used TWAS approaches, such as FUSION (Gusev et al., 2016) and PrediXcan (Gamazon et al., 2015), often use regularized regression or other selection criteria (e.g., “cis” selection) for the selection of SNPs, and then treat each intermediate as independent adopting a Bonferroni‐correction to account for inflated family‐wise error rate due to multiple comparison (Gamazon et al., 2015). Treating the gene expression data as independent is likely to result in an increase in false positives due to linkage and pleiotropy. Existing colocalization tests, such as fine‐mapping of gene sets (FOCUS) (Mancuso et al., 2019), linkage disequilibrium (LD)‐aware MR‐Egger (Barfield et al., 2018), summary data‐based Mendelian randomization (SMR) (Zhu et al., 2016), and multiple outcome probabilistic Mendelian randomization with Egger assumption (moPMR‐Egger) (Liu et al., 2021), offer a solution to handle linkage but cannot be easily scaled into a large number of intermediates.

In this paper, we introduce a new method within an IV framework, a Scalable Hier Archical Joint Analysis of Marginal summary data (SHA‐JAM). SHA‐JAM's goal is to discover and obtain an unbiased estimate for high dimensional intermediate data while leveraging GWAS genomic data that is potentially in linkage. This approach extends our previous work, hJAM, (Jiang et al., 2021) which is a hierarchical model for estimating the joint effect of multiple IVs on the outcome under the IV analysis framework. To enable efficient inference in a high‐dimensional setting, SHA‐JAM requires only SNP‐intermediate and SNP‐outcome summary statistics, and a reference genotype data set to estimate LD. We use a recently proposed variable selection algorithm, Sum of Single Effect (SuSiE) models (Wang et al., 2020), for selection in a two‐step process. First, to select the SNPs and estimate corresponding SNP‐intermediate associations. Second, to condition and select intermediates associated with the outcome. Across extensive simulation studies, SHA‐JAM exhibited an overall better performance, including higher area under the receiver operating characteristics (ROC) curve (AUC), lower mean square error (MSE) and better computation efficiency, when compared with existing approaches. To characterize the effect of metabolites and transcriptomics on prostate cancer risk, we applied SHA‐JAM on summary statistics from a large prostate cancer GWAS (79,194 cases and 61,112 controls) together with 30 tested metabolites and 1028 tested genes. We found that at least four highly correlated metabolites out of 30 tested metabolites and 55 out of 1028 tested genes showed effects on the risk of prostate cancer, respectively. Among the 55 genes, 37 have not been reported in previous TWAS but many showed biological relevance to prostate cancer in other studies.

## 2 METHODS

The hJAM approach is a form of IV analysis using marginal summary statistics to model the intermediates jointly to potentially address issues of pleiotropy (Jiang et al., 2021). To extend hJAM for high‐throughput experimental data, we developed SHA‐JAM by integrating the hJAM framework with a variable selection algorithm SuSiE (Wang et al., 2020) using sufficient statistics computed from GWAS summary data.

### 2.1 hJAM with individual level data

The IV analysis can be expressed as a two‐stage model when individual level data are available. Stage 1 models the outcome as a linear function of the genotypes:

(1) $$y=G\beta +e,\;e∼N(0,\;\sigma^{2}I_{K})$$

and stage 2 models the effect estimates $\beta {\;\in \;\mathbb{R}}^{K\times 1}$ from stage 1 as a function of a prior information matrix $\hat{A}{\in R}^{K\times M}$:

(2) $$\beta =\hat{A}\pi +\epsilon$$

respectively. The vector $\pi {\in \mathbb{R}}^{M\times 1}$ is the effect estimates of the intermediates on the outcome, that is, the association effects (Chen & Witte, 2007; Conti & Witte, 2003; Greenland, 2000b; Lewinger et al., 2007; Witte et al., 1994). Here, K and M denote the number of SNPs and intermediates, respectively, and $y\in R^{n\times 1}$ and $G{\in R}^{n\times K}$ denote the mean‐centered n‐individual outcome and genotype data, respectively. In this context, the weight matrix $\hat{A}$ contains the association estimates between the genetic variants and intermediates $X^{\prime }s$ and is computed using a different data set from the data used in Eq. (1). Each column of the matrix, $\hat{A}$, corresponds to a specific intermediate and the rows are SNPs associated with any intermediate and the elements within the matrix are the corresponding effect estimates from SNP‐intermediate combinations. In practice, the genetic variants to be included in $\hat{A}$ are those with a strong association with at least one of the intermediates to satisfy the relevance assumption in IV analysis. For example, in TWAS, SNPs in $\hat{A}$ are often chosen from those that are in the regulatory region of the genes of interests and explain partial variation in expression levels of these genes. In MR analysis that investigates the effect of multiple metabolites on the outcome, the $\hat{A}$ matrix will contain a set of SNPs that are significantly associated with at least one metabolite. With summary statistic data, as done in MR and TWAS analysis, we set ϵ=0 to enforce the no‐pleiotropy effect assumption as this parameter is unidentifiable in the current framework. Fitting the two‐stage model into a linear mixed model, we have

(3) $$y=G\hat{A}\pi +e$$

Overall, this analysis can be viewed as a two‐step approach in which step 1 is to construct the weight matrix $\hat{A}$ with the estimates between genetic variants and the intermediates, and step 2 estimates the effects of the intermediates on the outcome. When we have a large number of genetic variants and intermediates, we formulate the step of constructing the weight matrix as a fine‐mapping problem aiming to identify the SNPs (i.e., the rows for the matrix) associated with each intermediate (i.e., the columns for the matrix) (Supporting Information S1: Figure 1) and thus maximize prediction.

Given a weight matrix $\hat{A}$, we apply the Sum of Single Effect (SuSiE) regression (Wang et al., 2020) to select the intermediates in the hJAM framework (Eq. 3). SuSiE is a recently proposed approach for variable selection for highly correlated data with sparse detectable effects such as in fine mapping for GWAS (Wang et al., 2020), and offers computational efficient selection that benefit from a iterative Bayesian Stepwise selection (IBSS) algorithm, which is analogous to a forward selection. When individual level data (G and y) are available, we define $\hat{X}:=G\hat{A}$ and fit $\hat{X}$ as the covariates matrix in SuSiE. See Supporting Information S1: Supplementary Methods for details of fitting hJAM with SuSiE for selection of intermediates.

### 2.2 Two‐step SHA‐JAM with summary data

SHA‐JAM extends this selection algorithm when only summary data are available both for constructing the weight matrix and for inference on the intermediates.

#### 2.2.1 Step 1: Weight matrix construction

The weight matrix in Equations (2) and (3), $\hat{A}\in R^{K\times M}$, can be viewed as a prior information and it consists of the association estimates between K genetic variants and M intermediates. Individual entries in $\hat{A}$ can be computed from external data and $\hat{A}$ can be expressed as

$${\hat{A}}_{K\times M}=\left[\begin{array}{lll} {\hat{\alpha }}_{11} & \ldots & {\hat{\alpha }}_{1M}\\ \vdots & \ddots & \vdots \\ {\hat{\alpha }}_{K1} & \ldots & {\hat{\alpha }}_{\mathrm{KM}} \end{array}\right]$$

where ${\hat{\alpha }}_{\mathrm{km}}$ denotes the unbiased association estimate between the genetic variant k and the intermediate m. This is a reasonable assumption as these summary statistics are often provided from GWAS or other similar association studies which considered potential confounders such as population structure, as part of the standard analysis. In most situations the $\hat{A}$ matrix comes from an external sample, which is an independent sample from that providing the $\hat{b}$ (SNP–outcome) association statistics that we used in next step. When all entries in $\hat{A}$are the estimates of *marginal* association between a genetic variant and an intermediate, we refer to $\hat{A}$ as a marginal matrix, ${\hat{A}}_{\mathrm{marginal}}$. While ${\hat{A}}_{\mathrm{marginal}}$ (i.e., marginal statistics) may be used for these types of analyses, we have shown previously (Jiang et al., 2021) and in this paper that it is better to use joint estimates. These joint estimates can be obtained by applying the hJAM framework which we describe in our previous work (Jiang et al., 2021) and in Supporting Information S1: Figure 1. The original analysis performed to provide the marginal statistics from the external sample can vary but is often calculated using generalized linear models.

Here, we consider the situation in which we have a moderate or large number of intermediates and summary statistics for a large number of correlated SNPs. In this situation, we adopt the JAM framework and incorporate it with the SuSiE algorithm, called SuSiE JAM, to compose a weight matrix when only highly correlated summary data are available. Although the original paper presenting SuSiE (Wang et al., 2020) is developed for individual level data, an implementation of SuSiE with summary‐level data is also available in the susieR package, provided by Zou et al. (2022) This implementation uses sufficient statistics, including G′G, G′x, and x′x, to fit the single effect regression and obtain the posterior distribution. The sufficient statistics were computed using the JAM framework (see Supporting Information S1: Supplementary Methods).

#### 2.2.2 Step 2: Intermediate selection of omic effects

To select the intermediates with summary data, we apply the SuSiE algorithm with sufficient statistics for a second time and compose the plug‐in estimates using the hJAM variables, including ${\left(G\hat{A}\right)}^{\prime }\left(G\hat{A}\right),{\left(G\hat{A}\right)}^{\prime }y$ and $y^{\prime }y$. As described above, the predicted intermediates are computed using the weight matrix that is composed from external data, assuming no pleiotropy effects (Eq. 3). Thus, we can construct the plug‐ins of the predicted intermediates $\hat{X}$ using the weight matrix from step 1 as

(4) $${\hat{X}}^{\prime }\hat{X}={\left(G\hat{A}\right)}^{\prime }G\hat{A}={\hat{A}}^{\prime }G^{\prime }G\hat{A},$$

(5) $${\hat{X}}^{\prime }y={\left(G\hat{A}\right)}^{\prime }y={\hat{A}}^{\prime }G\prime y.$$

Note that composing the G′G, $G^{\prime }y$, and y′y are the same as in step 1 for composing the G′G, G′x, and x′x (see Supporting Information S1: Supplementary Methods). The LD structure of the GWAS for the association estimates between the SNPs and the outcome has to be the same as in step 1. Algorithm 1 shows the pseudo‐code for implementing step 2.

**Table:** 

**Algorithm 1:** Pseudoalgorithm for fitting SHA‐JAM with IBSS in intermediates selection
*Input data*: marginal effect estimates of SNP‐outcome relationship, $\hat{b}$, standard error of $\hat{b}$, $\mathrm{se}\left(\hat{b}\right),$ sample size of SNP‐outcome GWAS, $N_{G_{y}},$ effect allele frequencies $\mathrm{EA}F_{G_{y}},$ a reference individual‐level genotype $G_{R}$, and the weight matrix $\hat{A}$ *Input arguments*: the largest number of credible sets allowed, L, and hyperparameters $\sigma_{0}^{2}$ and $\sigma^{2}$. *Function required*: (1) $\text{SER\_sufficient\_statistics}(X^{\prime }X,X^{\prime }y;\sigma_{0}^{2},\sigma^{2})\to \left(\nu ,\mu_{1},\sigma_{1}\right)$ that computes the posterior function for $\pi_{l}$ under the SER model with sufficient statistics; (2) h_JAM_ $\left(\hat{b},\;\mathrm{se}(\hat{b}),\;N_{G_{y}},\;\mathrm{EA}F_{G_{y}},\;G_{R},\hat{\;A})\to ({\left(G\hat{A}\right)}^{\prime }(G\hat{A}),{\left(G\hat{A}\right)}^{\prime }y,y^{\prime }y\right)$ that computes the hJAM variables for function SER_sufficient_statistics. 1. Get hJAM variables: $\left({\left(G\hat{A}\right)}^{\prime }(G\hat{A}),{\left(G\hat{A}\right)}^{\prime }y,y^{\prime }y)\leftarrow \text{hJAM}(\hat{b},\;\mathrm{se}(\hat{b}),N_{G_{y}},\mathrm{MA}F_{G_{y}},\;G_{R},\;\hat{A}\right)$ For simplicity, we denote $\hat{X}≔G\hat{A}$ in the following text. 2. Initialize posterior means ${\overset{®}{\pi }}_{l}=0$, for l=1,…,L 3. Repeat 4. For l in 1,…,L do 5. ${\hat{X}\prime \overset{®}{r}}_{l}\leftarrow {\hat{X}}^{\prime }y-\hat{X}\prime \hat{X}\sum_{l^{\prime }\neq l}{\overset{®}{\pi }}_{l^{\prime }}$ 6. $\left(\nu ,\mu_{1},\sigma_{1}\right)\leftarrow \text{SER\_sufficient\_statistics}({\hat{X}}^{\prime }\hat{X},{\hat{X}}^{\prime }{\overset{®}{r}}_{l};\sigma_{0}^{2},\sigma^{2})$* 7. ${\overset{®}{\pi }}_{l}\leftarrow \nu_{l}\circ \mu_{1l}$ (∘ denotes the elementwise multiplication.) 8. Until convergence criterion satisfied 9. For m in 1,…,M do 10. $\left(\pi^{\left(m\right)},\text{PI}P_{m})\leftarrow {(\nu }_{1m},\mu_{11m},\sigma_{11m},\ldots ,\nu_{\mathrm{Lm}},\mu_{1\mathrm{Lm}},\sigma_{1\mathrm{Lm}}\right)$ Return π and PIP

*Note*: this algorithm is modified from Algorithm 1 in Wang et al. (2020). EAF, effect allele frequency.

* $\sigma_{0}^{2}$ and $\sigma^{2}$ could be updated in the fitting procedure which is provided by the SuSiE R package.

After we obtain the posterior $\hat{\pi }$ and **PIP** for M intermediate, we then construct the ρ‐level credible sets (default is 0.95). Within each credible set, we restrict the minimum absolute correlation coefficient between the included intermediates to a pre‐set value, such as 0.5.

### 2.3 Simulation study

#### 2.3.1 Simulation settings

**Figure 2:**
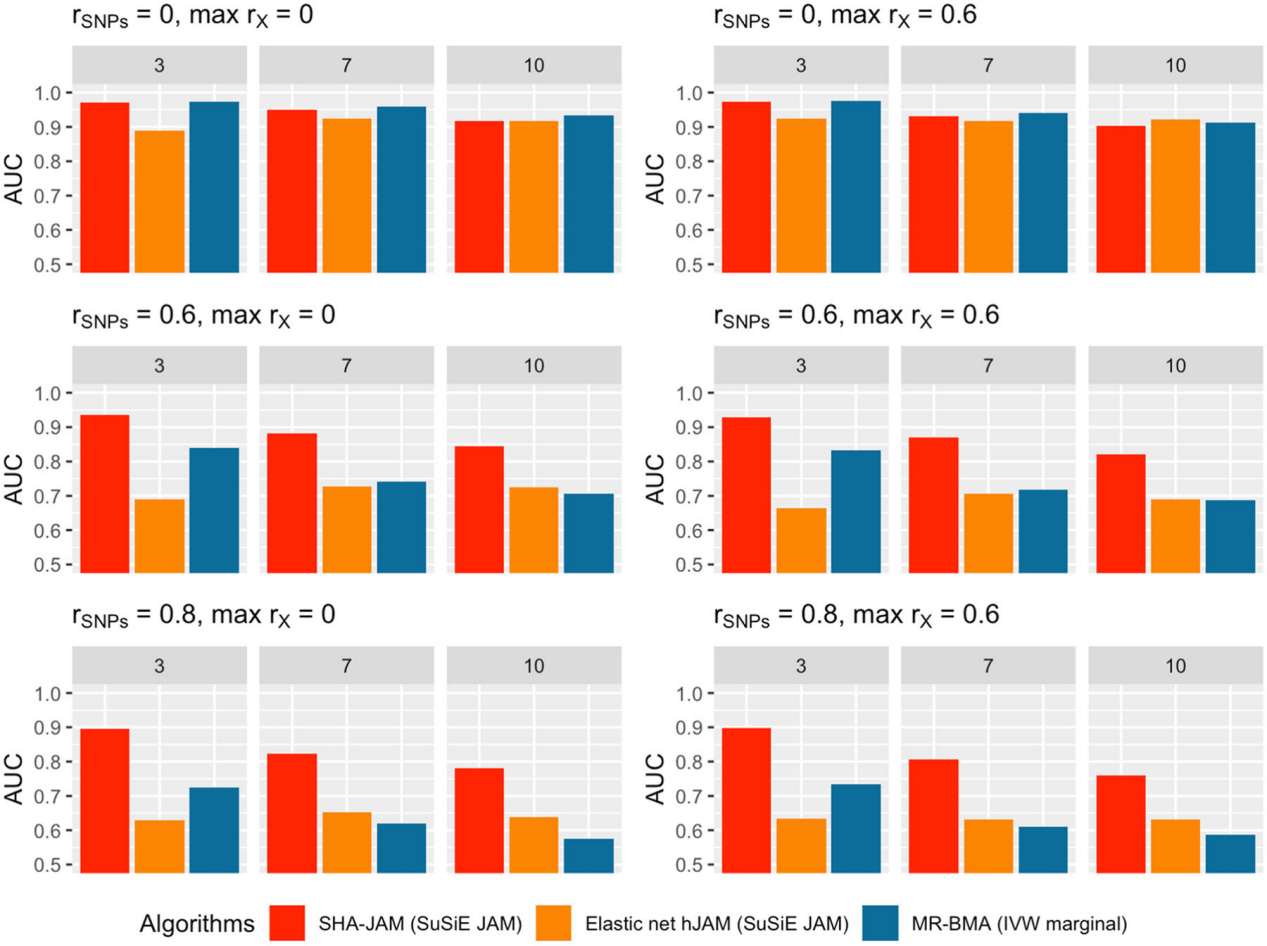
Intermediate selection: AUC comparison for SHA‐JAM, EN‐ hJAM, and MR‐BMA. Simulation results for three LD structures with different number of causal intermediates: 3, 7, and 10. The bar plots show the average AUC values. The algorithms are displayed as “Selection algorithm (weight matrix algorithm).” We used a pruned inverse‐variance weighted marginal matrix for MR‐BMA and SuSiE JAM weight matrix for SHA‐JAM and EN‐hJAM. For correlated SNP scenarios, we used a threshold of $r^{2}\leq 0.16$ to prune the genetic variants for the inverse‐variance weighted marginal matrix. The MR‐BMA was run with 500 iterations for the shotgun stochastic search.

For each replicate, we simulated three standardized individual genotype data (the LD reference panel $G_{R}$, the genotype in SNP‐intermediate study $G_{X}$, and the genotype in SNP‐outcome study $G_{Y}(K=300))$, an intermediate matrix X(M=50), and an outcome vector y. The LD structures and number of independent blocks ${(n}_{\text{SNP Blocks}}=10)$ were the same across the three G's. The effect allele frequency (EAF) of the SNPs were randomly drawn from a uniform distribution of (0.05, 0.3). Sample size of the three G's was $N_{G_{R}}=500$, $N_{G_{X}}=5000$, and $N_{G_{y}}=5000$, respectively. We have 30 SNPs per block and set three LD scenarios for each block: independent SNPs with $r_{\text{within block}}=0$, moderately correlated SNPs with $r_{\text{within block}}=0.6$, and highly correlated SNPs with $r_{\text{within block}}=0.8$ (Supporting Information S1: Figure 2). For intermediate m, 5 out of 300 SNPs were randomly picked as the causal SNPs and the effect size (i.e., column m of true weight matrix) of the 5 causal SNPs was defined by the correlation between the SNPs and the intermediate with total $r_{G,X_{m}}=0.1$. We simulated two correlation structures for the intermediate data: correlated intermediates with $\max\left(r_{X}\right)=0.6$ and independent intermediates with $\max\left(r_{X}\right)=0$ (Supporting Information S1: Figure 3). Among the 50 intermediates, we randomly set 0, 3, 7, and 10 intermediates to be causal with an effect size of π=0.3.

The set of summary statistics required were then generated from the individual level data (see Supporting Information S1: Supplementary Methods), including

(1) an effect estimates vector $\hat{b}$ and the corresponding $\mathrm{se}(\hat{b})$ from $G_{y}$ and y;
(2) a vector of the MAF of the SNPs from $G_{y}$;
(3) a weight matrix $\hat{A}$ from $G_{X}$ and X;
(4) an LD structure from $G_{R}$.

#### 2.3.2 Simulation analysis

We compare the performance of our approach, SHA‐JAM, with MR‐BMA (Zuber et al., 2020) and elastic net hJAM (EN‐hJAM). MR‐BMA is essentially an inverse‐variance weighted (IVW) linear regression that is implemented on the genetic associations with an outcome and the genetic associations with all intermediates (Burgess & Thompson, 2015). The intermediates selection by MR‐BMA then becomes variable selection in the linear model. It is formulated in a Bayesian framework which assigns uniformly independence prior to all intermediates and evaluates each model in space using closed‐form Bayes factors. Bayesian model averaging is applied since possible models are likely to be comparable. MR‐BMA draws posterior inference by the marginal inclusion probability (MIP), which is defined as the sum of the posterior probabilities for each intermediate across all possible models, and model‐averaged causal effects (MACE), which is defined as the average posterior mean for each intermediate over models. EN‐hJAM is another extension of hJAM where we first perform a Cholesky decomposition to simplify the likelihood of the summary statistics (Newcombe et al., 2016) and then implement the intermediate selection with elastic net regularized regression (Zou & Hastie, 2005) (see Supporting Information S1: Supplementary Methods).

Since different selection algorithms require different weight matrices, we constructed two types of weight matrices. For SHA‐JAM and EN‐hJAM, SuSiE JAM is applied to construct the weight matrix. For selecting the SNPs, we first pruned the summary data to exclude highly correlated SNPs with an absolute pairwise correlation exceeding 0.9. We then fit the marginal summary statistics with SuSiE hJAM to obtain the posterior mean as the elements in the weight matrix. For MR‐BMA, following the original paper (Zuber et al., 2020) we pruned the summary data to only include independent SNPs (i.e., an absolute pairwise correlation less than 0.4) and applied an IVW matrix to the marginal summary data. To mimic realistic applications, we simulated individual‐level genotypes and intermediates (i.e., $G_{X}$ and X; sample size $N_{G_{X}}=5000$) to obtain the SNP‐intermediate summary statistics. As a sensitivity analysis, we assessed the performance of different weight matrices as well as the performance of intermediates selection using these weight matrices. We also randomly subset the large data into a relatively smaller sample for a weight matrix constructed with individual level data with a smaller sample ($N_{\text{subset}}=500$) and assessed the impact of the sample size of the data for computing the weight matrix on SHA‐JAM.

In the intermediate selection step for SHA‐JAM, we set the coverage of credible set as 0.95, the minimum absolute correlation between the variants in each credible set as 0.5 and the largest number of credible sets to be 10. We conducted additional sensitivity analyses to evaluate the impact of different parameter settings in SHA‐JAM on the performance of intermediate selection, including setting the credible set coverage to 0.90 instead of 0.95, varying the minimum absolute correlation in each credible set, and varying the maximum number of credible sets. For EN‐hJAM, we used the glmnet package and 10‐fold cross‐validation to tune the shrinkage parameters using the lambda.1se which selects a model such that the mean cross‐validation error is within one standard error of the minimum. For the sake of running time, we set 500 iterations for the shotgun stochastic search with a prior probability of 0.1 for MR‐BMA. A sensitivity analysis was performed across five replicates with 100,000 iterations for the shotgun stochastic search to compare. In MR‐BMA, the minimum and maximum number of risk factors per model was set to be one and five more intermediates than the true number of causal intermediates to facilitate model exploration with more than the number of true causal intermediates while minimizing computational expenses.

To evaluate the performance of different selection algorithms, we first checked the false‐positive rate for scenarios in which the intermediate effect is null. Then, for scenarios with at least one non‐zero intermediate effect, we compared the AUC, MSE (i.e., $\frac{1}{M}\sum_{i=1}^{M}{\left({\hat{\pi }}_{i}-\pi_{i}\right)}^{2}$) and absolute bias (i.e., $\frac{1}{M}\sum_{i=1}^{M}\left|{\hat{\pi }}_{i}-\pi_{i}\right|$) of the selected estimates. We also checked the averaged specificity, sensitivity, and positive predicted value (PPV) of selected intermediates across all simulation scenarios. The true positives are defined as the true non‐zero intermediate being selected by the model: included in the credible sets for SHA‐JAM, non‐zero effect for EN‐hJAM, and a marginal probability larger than 0.2 for MR‐BMA. Additionally, we evaluated the validity of credible set in SHA‐JAM with the fraction of causal intermediates in a credible set at 90% and 95% coverage, averaged over all credible sets. We also explored an alternative approach of intermediate selection for SHA‐JAM in which intermediates with posterior inclusion probability greater than a certain threshold are defined as having non‐zero effects and those below the threshold are set to zero. All simulation studies were performed in R version 3.6.0.

### 2.4 Data examples

#### 2.4.1 Selecting metabolites associated with prostate cancer risk

Some studies have found suggestive associations between reduced prostate cancer risk among men with low cholesterol (Heir et al., 2016; Platz et al., 2009), triglycerides (TG) (Arthur et al., 2016; Van Hemelrijck et al., 2011), and fatty acid levels (Newcomer et al., 2001). However, univariate MR analyses between metabolites and prostate cancer risk have found inconsistent results regarding the causal nature of these associations (Adams et al., 2019; Bull et al., 2016; Khankari et al., 2016; Orho‐Melander et al., 2018; Pierce et al., 2018). This may be in part due to these previous MR analyses only including a few metabolites and modeling them individually, thus ignoring the high correlation between the intermediates.

To gain a better understanding of the effects of metabolites on prostate cancer risk, we applied SHA‐JAM, EN‐hJAM, and MR‐BMA to select metabolites likely to be causally associated with prostate cancer risk. The weight $\hat{A}$ matrix is constructed using summary data from Zuber et al. (2020) (https://github.com/verena=zuber/demo_AMD), which includes 150 significant metabolites‐associated SNPs from a large‐scale meta‐analysis of the Global Lipids Genetics Consortium (GLGC) (Willer et al., 2013). Zuber et al. (2020) tested 118 metabolites and 69 metabolites were excluded since they were highly correlated with other metabolites ($\left|r_{\text{Metabolites}}\right|\geq 0.985$) while 19 were excluded that had no univariate‐significant associated genetic variants in the summary data. After the exclusion, there were 30 candidate metabolites (Supporting Information S1: Table 6). The $\hat{\beta }$ and $\mathrm{se}(\hat{\beta })$ of the genetic variants that are identified in ${\hat{A}}_{\text{marginal}}$ matrix were taken from a large prostate cancer GWAS with 79,194 prostate cancer cases and 61,112 controls (Schumacher et al., 2018). The study population in the GWASs are all European ancestry. Thus, the European ancestry genotype data from 1000 Genome Project (Auton et al., 2015) was used as the reference panel for extracting the LD structure ($N_{1000\text{Genome}}$ = 503). To harmonize all the data, we further excluded four SNPs which were missing in the prostate cancer GWAS (rs2652834, rs9930333, rs1998013, and rs894210) and one SNP with no variation in the 1000 Genome data (rs2290547). We also identified two highly correlated SNPs (rs11246602 and rs12226802, |$r_{\text{SNPs}}|=0.97$) with same effect on the metabolites and excluded one randomly. MR‐BMA requires a model checking step to examine outliers. Four SNPs were further excluded after we investigated the model fit for MR‐BMA using Cook's distance and *q*‐statistics identified potential outliers among the best models with posterior probability higher than 0.02 and found four invalid IVs. The genetic variant rs2710642 in the *EHBP1* gene was excluded since it showed a large value in both Cook's distance and *q*‐statistics in models with the highest posterior probabilities (Supporting Information S1: Figure 11a,b). This gene was reported to be strongly associated with prostate cancer risk in European‐ancestry subjects (Ao et al., 2015). We re‐ran the analysis after excluding rs2710642 and identified three more influential variants: rs261342 with large Cook's distance and rs205262 and rs267733 with large *q*‐statistics (Supporting Information S1: Figure 11c,d). After the exclusion of the aforementioned SNPs from the 150 significant metabolites‐associated SNPs in Zuber et al. (2020), a total of K=140 genetic variants remained in the analysis. Four metabolites (M=26) with no genome‐wide significant $\left(P\;<\;5\times 10^{-8}\right)$ associated SNPs remaining in the analysis were then removed.

For SHA‐JAM, we set the minimum correlation of the variants within one credible set (level = 0.9) as 0.6 and the maximum number of credible sets as 5. As a comparison, we applied the inverse variance weighted marginal ${\hat{A}}_{\text{marginal}}$ and $\hat{\beta }$ to MR‐BMA to select the causal metabolites of prostate cancer risk. We set the prior probability of 0.1, a minimum and maximum size of 1 and 12 metabolites, respectively, per model, and with 100,000 iterations in the shotgun stochastic search, as suggested by the original paper (Zuber et al., 2020).

### 2.5 Selecting the genes associated with prostate cancer risk

In the second real data example, we used significant prostate tissue expression quantitative trait loci (eQTLs)‐gene pairs with 174,876 eQTLs and 3947 genes that were identified based on permutations by the GTEx Project (Lonsdale et al., 2013) (https://storage.googleapis.com/gtex_analysis_v7/single_tissue_eqtl_data/GTEx_Analysis_v7_eQTL.tar.gz) (Lonsdale et al., 2013), and applied our analyses independently on each chromosome (Supporting Information S1: Table 8). We used prostate tissue only as it has been shown that the gene expression in prostate tissue is the most relevant to the risk of prostate cancer (Mancuso et al., 2018). The coefficients in the marginal ${\hat{A}}_{\text{marginal}}$ matrix were obtained from GTEx analysis v7 (https://storage.googleapis.com/gtex_analysis_v7/single_tissue_eqtl_data/GTEx_Analysis_v7_eQTL_all_associations.tar.gz). We pre‐pruned the data to exclude SNPs with pairwise correlation coefficient |r|>0.9, which reduced the number of eQTLs and genes to 9894 and 1028, respectively (Supporting Information S1: Table 8). Marginal GWAS summary coefficients were obtained from GTEx (Lonsdale et al., 2013) from 132 male participants and used to estimate joint effects using SuSiE JAM. The corresponding prostate cancer summary data for these variants were obtained from the same prostate cancer GWAS as in the first data example (Schumacher et al., 2018). Similarly, we used the European‐ancestry population in the 1000 Genome Project (Auton et al., 2015) as the reference genotype data.

We then applied SuSiE JAM on the marginal ${\hat{A}}_{\text{marginal}}$ to compute the posterior mean and obtained the joint $\hat{A}$. Genes with all elements equal to zero in the joint $\hat{A}$ were removed (i.e., genes with no corresponding SNP effects). Supporting Information S1: Table 8 shows the dimensions of the final $\hat{A}$ matrix for each chromosome. The association coefficients between the identified genetic variants and prostate cancer, $\hat{\beta }$ and $\mathrm{se}(\hat{\beta })$, were obtained from the same prostate cancer GWAS as in the first data example (Schumacher et al., 2018). We used the European‐ancestry population in the 1000 Genome Project (Auton et al., 2015) as the reference genotype data. For SHA‐JAM, we set the minimum correlation of the variants within one credible set (level = 0.95) as 0.6 and the maximum number of credible sets as 1/5 of the total genes being included per chromosome.

## 3 RESULTS

### 3.1 SHA‐JAM outperforms current multi‐variate approaches in simulations

The overall average AUC across all scenarios was 0.883, 0.751, and 0.781 for SHA‐JAM, EN‐hJAM, and MR‐BMA, respectively. SHA‐JAM consistently outperformed MR‐BMA and EN‐hJAM, regardless of the correlation structure of the intermediates in correlated SNPs scenarios ($r_{\text{within block}}=0.6$ and $r_{\text{within block}}=0.8$) (Figure 2). This enhanced performance of SHA‐JAM over MR‐BMA is most likely due to the flexibility of hJAM to handle correlated SNPs in the weight matrix construction and subsequently in the selection of intermediates. In contrast, MR‐BMA requires independent SNPs. Both EN‐hJAM and MR‐BMA showed a relatively consistent average AUC across different number of causal intermediates while SHA‐JAM performed better in scenarios with fewer causal intermediates—a finding also found in the original report for SuSiE with individual‐level data (Wang et al., 2020). The performance of each algorithm did not differ between the correlated and independent intermediates scenarios. Under the extreme scenarios where more than 10 intermediates (out of 50) are causal, EN‐hJAM has similar performance to SHA‐JAM in terms of AUC and slightly better performance when there is no SNP correlation (Supporting Information S1: Figure 6). When no causal intermediate existed, EN‐hJAM and SHA‐JAM identified almost zero intermediates across all scenarios while MR‐BMA identified an average number of 0.747 (1.49%) and 2.866 (5.73%) intermediates across scenarios with independent and correlated SNPs, respectively (Table 1). The higher false‐positive rate for MR‐BMA likely due to the approach requiring at least one risk factor to be included in the model in each step of the estimation procedure. When no causal intermediates truly exist, such an assumption will clearly lead to false positives.

**Table 1:** Intermediate selection: Average number of false positives for SHA‐JAM, EN‐ hJAM, and MR‐BMA.

$r_{\mathrm{within block}}$ a	$\max\;r_{X}$ b	Selection algorithm		
		SHA‐JAM	EN‐hJAM	MR‐BMA
0	0	0.007 (0.01%)	0 (0%)	0.649 (1.30%)
	0.6	0 (0%)	0 (0%)	0.845 (1.69%)
0.6	0	0.006 (0.01%)	0 (0%)	3.520 (7.04%)
	0.6	0.010 (0.02%)	0 (0%)	3.345 (6.69%)
0.8	0	0.008 (0.02%)	0 (0%)	2.335 (4.67%)
	0.6	0.001 (0%)	0 (0%)	2.430 (4.86%)

^a^ Correlation within block of ***G*** 's.

^b^ Maximum correlation coefficient between intermediates **X**. This table shows the average number of false positives out of 50 noncausal intermediates. A positive selection was defined as a marginal inclusion probability >0.2 for MR‐BMA, a non‐zero coefficient for EN‐hJAM, and selection in the credible sets for SHA‐JAM, respectively.

With respect to the MSE, when causal intermediates existed, SHA‐JAM had the lowest MSE followed by MR‐BMA across almost all scenarios. The exception was a low MSE for MR‐BMA in scenarios with simulated independent SNPs and intermediates and a larger number of causal intermediates (Table 2). For the correlated SNP scenarios using MR‐BMA, the larger MSEs appeared to be driven by the bias of the estimates (Supporting Information S1: Table 1), not necessarily an increase in the variance. When no causal intermediate existed, EN‐hJAM showed the lowest MSE across all scenarios, again driven by the bias introduced by the regularization of estimates in the elastic‐net model (Zou & Hastie, 2005).

**Table 2:** Intermediate estimation: mean‐squared errors (MSE) of the intermediate for SHA‐JAM, EN‐hJAM, and MR‐BMA.

$r_{\mathrm{within block}}$ a	$\max\;r_{X}$ b	Selection algorithm	Number of causal intermediates			
			0	3	7	10
0	0	SHA‐JAM	0.001	**0.025**	**0.097**	0.198
		EN‐hJAM	**0**	0.123	0.237	0.336
		MR‐BMA	0.001	0.031	0.099	**0.184**
0	0.6	SHA‐JAM	0.001	**0.023**	**0.115**	**0.226**
		EN‐hJAM	**0**	0.112	0.259	0.328
		MR‐BMA	0.003	0.031	0.128	0.232
0.6	0	SHA‐JAM	0.013	**0.046**	**0.209**	**0.388**
		EN‐hJAM	**0**	0.220	0.476	0.670
		MR‐BMA	0.026	0.163	0.479	0.758
0.6	0.6	SHA‐JAM	0.003	**0.050**	**0.218**	**0.435**
		EN‐hJAM	**0**	0.230	0.496	0.708
		MR‐BMA	0.027	0.163	0.518	0.832
0.8	0	SHA‐JAM	0.004	**0.070**	**0.292**	**0.618**
		EN‐hJAM	**0**	0.238	0.529	0.788
		MR‐BMA	0.022	0.262	0.767	1.175
0.8	0.6	SHA‐JAM	0.019	**0.067**	**0.432**	**0.601**
		EN‐hJAM	**0**	0.237	0.541	0.777
		MR‐BMA	0.018	0.251	0.765	1.145

^a^ Correlation within block of ***G*** 's.

^b^ Maximum correlation coefficient between intermediates ***X***. We showed the mean‐squared error (MSE) of each selection algorithm with best performed $\hat{A}$ matrix. The lowest MSE was bolded for each scenario. We used pruned inverse‐variance weighted marginal ${\hat{A}}_{\mathrm{marginal}}$ for MR‐BMA, SuSiE JAM for SHA‐JAM $\hat{A}$ and EN‐ hJAM.

For these simulations, all of the three methods perform well in terms of sensitivity and PPV with slightly better performance for SHA‐JAM while SHA‐JAM performs better in specificity (Supporting Information S1: Table 2). SHA‐JAM has an average specificity of 0.77 while MR‐BMA and EN‐hJAM have 0.63 and 0.52, respectively. For sensitivity, SHA‐JAM has an average sensitivity of 0.99 while MR‐BMA and EN‐hJAM have 0.94 and 0.98, respectively. For PPV, SHA‐JAM has an average sensitivity of 0.96 while MR‐BMA and EN‐hJAM have 0.94 and 0.93, respectively. When we changed the coverage of credible set from 0.95 to 0.90, the sensitivity, specificity, and positive predictive value did not change substantially (Supporting Information S1: Table 3). Credible sets are approximately calibrated for the fraction of causal intermediates with a given credible set (see Supporting Information S1: Figure 10).

We conducted sensitivity analyses to assess the performance of different weight matrices as well as selecting the intermediates using these different weight matrices. The estimates from SuSiE with individual data and SuSiE JAM using summary data showed low averaged MSEs and low bias even in high correlated SNPs scenario (Supporting Information S1: Table 4). We demonstrated that a weight matrix constructed using SuSiE JAM with summary statistics resulted in equivalent AUCs as comparing to constructing a weight matrix using SuSiE with individual level data. These results hold across all scenarios (Supporting Information S1: Figure 4). As expected, the AUC is the highest when the weight matrix is defined by the true SNP‐intermediate effects, and AUC decreases when the sample size of the external data for constructing the weight matrix decreases (Supporting Information S1: Figure 5).

In addition to the default parameter settings we used for SHA‐JAM (i.e., the same parameter settings used in the main simulation analysis shown in Tables 1 and 2), we varied the settings for two parameters, the maximum number of credible sets (denoted as “*L*”) and the minimum absolute correlation allowed in a credible set (denoted as “|*r*|”), to explore their impact on SHA‐JAM's performance under different causal architectures. As shown in Supporting Information S1: Figures 7 and 8, AUC would drop substantially when *L* is less than the number of true causal intermediates (model misspecification). When *L* exceeds the number of true causal intermediates, increasing *L* further has very limited impact on AUC as the model has already been saturated (Wang et al., 2020). Furthermore, changing |*r*| does not impact AUC when *L* is correctly specified (i.e., greater or equal to the number of true causals) in the simulated data. We also examined performance of intermediate selection using an alternative approach of defining positives in SHA‐JAM: intermediates with posterior inclusion probabilities (PIP) exceeds a pre‐specified cutoff are defined as selected, and others as not selected. As shown in Supporting Information S1: Figure 9, setting a threshold of PIP ≥ 0.8 had very similar performance compared to the results that are based on SuSiE 95% credible sets.

While the runtime increases as the intermediates or the complexity between the SNPs increases, SHA‐JAM and EN‐hJAM were about 300 and 150 times faster than MR‐BMA, respectively (Supporting Information S1: Table 5). As the computation time increases with the maximum number of iterations in the shotgun stochastic search increases for MR‐BMA, we set the maximum number of iterations to 500 in assessing runtime for the sake of a large number of replications. When the maximum iterations was set to 100,000, as done in the data example for the original MR‐BMA paper (Zuber et al., 2020), the approach takes about 2 h to complete with 50 candidate intermediates. Comparison of results from a few replicates in the simulations showed little difference between inference with 100,000 iterations versus 500.

### 3.2 SHA‐JAM identifies metabolites with putative causal effect on prostate cancer risk

Results showed that SHA‐JAM identifies four metabolites in one credible set (level = 0.96), demonstrating a positive association with prostate cancer risk (Table 3). The four metabolites are total cholesterol in small LDL (S.LDL.C, posterior inclusion probability, PIP = 0.39), total cholesterol in IDL (IDL.C, PIP = 0.14), total cholesterol in LDL (LDL.C, PIP = 0.20), and serum total cholesterol (Serum.C, PIP = 0.22). The minimum and mean absolute correlation between the four metabolites was 0.93 and 0.96, respectively. Results from MR‐BMA showed no intermediates with MIP > 0.2, although the top three metabolites in MR‐BMA (0.070 < MIP < 0.119) correspond with the SHA‐JAM results. IDL.C, which showed the lowest PIP in the credible set of SHA‐JAM, ranked as the top 7th metabolite with MR‐BMA (MIP = 0.056). Interestingly, the selected metabolites in the top four best models from MR‐BMA (Supporting Information S1: Table 7) correspond to the four metabolites selected with SHA‐JAM. Supporting Information S1: Table 8 shows the comparison of PIP/MIP and effect estimates for the analyses with and without the influential genetic variants. SHA‐JAM shows more consistent results suggesting potential limited influence of outliers or invalid instrument variables (Supporting Information S1: Table 8 and Supporting Information S1: Figure 12).

**Table 3:** Selecting causal metabolites for risk of prostate cancer.

Selection algorithm	Selected metabolites^a^	PIP/MIP^b^	Coefficient^c^	Runtime
With potential influential genetic variants (*K* = 144, *M* = 30)				
SHA‐JAM	S.LDL.C	0.388	0.05	0.146 s
	IDL.C	0.263	0.03	
	LDL.C	0.243	0.029	
	Serum.C	0.066	0.008	
MR‐BMA	M.VLDL.TG	0.093	−0.032	7.470 min
	S.VLDL.TG	0.091	0.028	
	IDL.C	0.089	0.009	
	Serum.C	0.078	0.009	
Without potential influential genetic variants (*K* = 140, *M* = 26)				
SHA‐JAM	S.LDL.C	0.393	0.038	0.026 s
	Serum.C	0.221	0.021	
	LDL.C	0.196	0.017	
	IDL.C	0.142	0.012	
MR‐BMA	Serum.C	0.119	0.017	6.022 min
	S.LDL.C	0.073	0.005	
	IDL.C	0.07	0.005	
	M.VLDL.TG	0.068	−0.011	

*Note*: EN‐hJAM selected no intermediates in the analysis.

Abbreviations: CS, credible set; IDL.C, total cholesterol in IDL; LDL.C, total cholesterol in LDL; MIP, marginal inclusion probability; M.VLDL.TG, triglycerides in medium VLDL; PIP, posterior inclusion probability; S.LDL.C, total cholesterol in small LDL; Serum.C, serum total cholesterol; S.VLDL.TG, triglycerides in small VLDL.

^a^ For the selected metabolites column, SHA‐JAM reports the metabolites that were selected in the credible sets and MR‐BMA reports the top four ranked metabolites since none had MIP > 0.2.

^b^ For the inclusion probability column, SHA‐JAM and MR‐BMA report the posterior inclusion probability and marginal inclusion probability, respectively.

^c^ For the coefficient column, SHA‐JAM and MR‐BMA display the posterior means and model‐averaged causal effects (MACE), respectively.

### 3.3 SHA‐JAM identifies genes associated with prostate cancer risk

In total, the SHA‐JAM analysis resulted in a total of 45 credible sets with a level of 0.95 and 55 genes (Supporting Information S1: Table 8). Overall, there is about a fourfold increase in the chance of reported evidence (Piñero et al., 2020) of gene‐PCa associations for genes that are selected in SHA‐JAM credible sets, compared to genes that were included in the analysis but not selected by SHA‐JAM. Among the 45 identified credible sets, 38 contained exactly one gene (Table 4), 6 contained two highly correlated genes (average correlation coefficient r=0.932) (Supporting Information S1: Table 9), and 1 contained five perfectly correlated genes (correlation coefficient r=1) (Supporting Information S1: Table 9). Moreover, the genes in each credible set were in the same region and contained the same eQTLs.

**Table 4:** Selected candidate genes for prostate cancer risk by SHA‐JAM (credible sets that contain only one gene).

Chromosome	Gene name	Gene type	Posterior mean	PIP	Reference
1	NUCKS1	*protein coding*	0.166	0.992	Lonsdale et al. (2013); Wu et al. (2019)
2	ALS2CR12	*protein coding*	−0.173	1	Wu et al. (2019)
2	GGCX	*protein coding*	−0.196	1	Lonsdale et al. (2013); Wu et al. (2019)
2	MLPH	*protein coding*	−0.312	1	Lonsdale et al. (2013); Wu et al. (2019)
2	AC007879.2	*lincRNA*	0.103	1	Stram et al. (2003)
2	SPET2	*protein coding*	−0.139	0.998	Wang et al. (2015)
3	CHMP2B	*protein coding*	0.443	1	Lonsdale et al. (2013); Wu et al. (2019)
3	RP11‐469J4.3	*lincRNA*	−0.140	1	
3	WDR52	*protein coding*	0.218	1	Lonsdale et al. (2013); Wu et al. (2019)
3	RP11‐446H18.1	*pseudogene*	0.129	1	
3	ACPP	*protein coding*	−0.221	0.999	Suburu and Chen (2012)
5	CTD‐3080P12.3	*antisense*	−0.045	0.997	Bhattacharya et al. (2020)
5	RP11‐184E9.2	*lincRNA*	0.038	0.973	
6	RGS17	*protein coding*	0.212	1	Lonsdale et al. (2013); Wu et al. (2019)
6	SESN1	*protein coding*	0.200	1	Lonsdale et al. (2013)
6	WTAP	*protein coding*	−0.143	1	Wu et al. (2019)
6	L3MBTL3	*protein coding*	−0.068	1	Gudbjartsson et al. (2008); Tibshirani et al. (2016); Vujkovic et al. (2020)
6	C6orf164	*protein coding*	0.097	0.966	Conti et al. (2021)
7	HOTAIRM1	*antisense*	0.065	0.999	Boudreau et al. (2008)
7	RP4‐607J23.2	*antisense*	−0.105	0.990	Wu et al. (2019)
7	TMEM184A	*protein coding*	0.153	0.968	Bansal et al. (2012)
9	CBWD6	*protein coding*	−0.010	1	
10	MSMB	*protein coding*	−0.762	1	Wu et al. (2019)
10	AGAP4	*protein coding*	−0.077	1	Kraft et al. (2005)
10	MGMT	*protein coding*	0.046	0.995	Kok et al. (2011)
10	RP11‐18I14.10	*processed transcript*	0.581	0.993	
11	RP11‐554A11.9	*antisense*	0.101	1	Wu et al. (2019)
11	MMP7	*protein coding*	0.188	1	Lonsdale et al. (2013); Wu et al. (2019)
11	RAD9A	*protein coding*	0.197	1	Wu et al. (2019)
12	RP1‐228P16.1	*pseudogene*	−0.091	1	
12	TUBA1A	*protein coding*	−3.598	1	Chen et al. (2022)
17	RP11‐115K3.1	*antisense*	0.523	1	Wu et al. (2019)
17	LRRC46	*protein coding*	0.105	0.995	Tang et al. (2021)
17	TRPV3	*protein coding*	−0.086	0.982	Lee et al. (2016)
18	RAB27B	*protein coding*	−4.223	0.995	Wayner et al. (2012)
19	ZNF571‐AS1	*antisense*	0.105	0.975	Taylor & Tibshirani (2015)
20	ABHD16B	*protein coding*	−6.385	0.995	Wainberg et al. (2019)
22	TBX1	*protein coding*	−0.072	1	Lonsdale et al. (2013); Wu et al. (2019)

*Note*: This table includes the genes from the credible sets that contained only one gene. There are 10 such credible sets in total.

Abbreviations: PIP, posterior inclusion probability.

In addition, we compared our results with two large‐scale TWAS for prostate cancer (Mancuso et al., 2018; Wu et al., 2019) that reported 109 and 137 noteworthy genes, respectively. Since we only included the genes which have significant associated eQTLs in the GTEx prostate tissue summary data, some previous reported genes in prostate cancer risk were not included in our analysis, such as *HNF1B* (Eeles et al., 2008; Emami et al., 2019). The numbers of overlapping genes between ours and Mancuso et al. (2018) and Wu et al. (2019) are 20 and 24, respectively. We were able to replicate most of the overlapping genes (18 out of 20/24 genes) and discovered additional 37 genes that have not been previously reported (Table 4 and Supporting Information S1: Table 9). For discovered genes that have not been reported in previous TWASs, we found 16 that showed biological relevance to prostate cancer in other studies (Table 4).

## 4 DISCUSSION

In this paper, we propose SHA‐JAM, a scalable hierarchical approach for joint analysis with summary statistics from high‐dimensional omics data. SHA‐JAM is an extension of hJAM, an approach we previously introduced for multivariable IV analysis that can be applied to MR or TWAS when only summary statistics is available. We demonstrate the flexibility of the hJAM method within an IV framework to model multiple intermediates with numerous SNPs and extend the method in a computationally efficient manner to high‐dimensional omic data in SHA‐JAM to perform agnostic selection of both the instruments from highly correlated SNP data and intermediates. This makes SHA‐JAM ideally suited for the analysis of numerous omic data types with available summary statistics, including transcriptomics, proteomics, metabolomics, and microbiome data. With a potential for such a broad application, care must be taken when interpreting the final estimates of association for corresponding intermediates. Only when all assumptions of the underlying IV analysis can be confirmed can any effect estimate be interpreted as “causal.” While incorporating genome‐wide SNP information and conditioning on multiple intermediates allows SHA‐JAM to account for linkage and potential pleiotropy, given the uncertainty in confidently concluding that all assumptions are valid in many situations with omic data, we believe it is most appropriate to avoid concluding any definitive causal relationships and interpret the effects as “possibly causal” or simply “associated.”

In real‐life applications, setting the parameters in SHA‐JAM (i.e., maximum number of credible sets “*L*” and minimum absolute correlation between intermediates “|*r*|”) might need a case‐by‐case evaluation. The maximum number of significant intermediates that we can identify can be based on sample sizes, correlation between SNPs, correlation between intermediates, and the type of outcome. We recommend setting *L* to a value at least greater than the number of intermediates that are marginally statistically significant, and |*r*| to a moderately high value (|*r*| = 0.5 as this performed well in our simulations and is the default value in native SuSiE algorithm). In cases when deciding an optimal value for |*r*| is difficult, an alternative approach is to select intermediates based on PIP of each intermediate in the model. Since |*r*| is an “ad‐hoc” parameter in the native SuSiE algorithm and it is only involved in forming credible sets, the value for |*r*| is independent of the posterior mean and posterior inclusion probabilities for each intermediate. In our simulations, we demonstrated that setting a threshold of PIP ≥ 0.8 had very similar intermediate selection performance compared to the results that are based on SuSiE 95% credible sets (Supporting Information S1: Figure 9).

Though SHA‐JAM is developed based on continuous intermediates and continuous outcome, it can also be applied to summary statistics from an analysis with binary outcomes. Given that the intermediates are well‐imputed with an appropriate weight matrix, to identify intermediates that are statistically associated with the outcome is similar to a fine‐mapping problem. The original joint analysis of marginal summary statistics (JAM) (Newcombe et al., 2016) noted a plausible transformation of logistic effects to linear effects for binary‐outcome studies. In the multi‐population fine‐mapping using hierarchical JAM (Shen et al., 2024), it has been shown that summary statistics in terms of log odds ratios yields similar fine‐mapping performance to summary statistics in terms of linear effects when statistical power is approximately similar. Therefore, applying SHA‐JAM to binary outcomes is considered feasible and appropriate, provided the intermediates are accurately imputed with a well‐constructed weight matrix.

We highlight the importance of the weight matrix construction for SHA‐JAM, as well as all MR and TWAS approaches. Construction of the weight matrix, including selecting SNPs truly associated with the intermediate and estimating corresponding associations, is essentially a fine‐mapping problem for intermediates. An optimal weight matrix can improve the performance of any of these approaches. We have provided different ways to construct the weight matrix under different situations, including the commonly used approaches of either marginal summary statistics or elastic net coefficients (Zou & Hastie, 2005) selected with individual genotype data and a novel approach using joint summary statistics obtained by converting marginal estimates using JAM (Newcombe et al., 2016). Genetic variants included in the marginal summary statistics matrix are first identified by univariate linear or logistic regression between the genetic variant and the intermediates often using a Bonferroni correction to account for multiple comparisons. Additional pruning steps, either statistically or biologically, are commonly applied to the set of the univariately significant genetic variants to produce the final set of genetic variants, of which the estimates in the weight matrix are the corresponding univariate regression coefficients. MR approaches use the inverse variance weighted (IVW) marginal summary statistics and require the genetic variants to be independent (Zuber et al., 2020). Construction of an elastic net matrix is implemented by PrediXcan (Gamazon et al., 2015) to estimate the effects of SNPs on gene expression levels in individual‐level genotype data. In contrast to these approaches, hJAM uses a joint summary statistics matrix for the same set of genetic variants included in the marginal matrix. These joint estimates are obtained through the JAM framework (Newcombe et al., 2016) by jointly analyzing the marginal summary statistics of multiple genetic variants (Jiang et al., 2021). Our previous study found univariate intermediate models, such as IVW MR and PrediXcan, subject to inflated type I error when another correlated intermediate also contributes to the risk of the outcome (i.e., pleiotropy effect exists) (Jiang et al., 2021). In the presence of numerous highly correlated genetic variants, we introduced SuSiE JAM as the first step in SHA‐JAM for weight matrix construction by adapting SuSiE within the JAM framework. This approach showed good performance in simulations and for our application in constructing the weight matrix with the highly correlated eQTLs and gene expression data. If individual‐level data are available and of sufficient sample size, SuSiE might be a better solution for constructing the weight matrix. Regarding the ad hoc pruning procedure, all algorithms for handling correlated data are limited in addressing very highly correlated variables. While the SuSiE algorithm allows us to push the limits, a preprocessing step using priority pruner to select the candidate intermediates in the data examples was used.

Specifically, whereas MR and TWAS often limit the number of SNPs to either previously identified SNPs or those in ‘cis’, SHA‐JAM can agnostically select SNPs as instruments potentially across the genome with proper pruning to obtain estimates for the corresponding SNP‐intermediate associations. As demonstrated in Panyard et al. (2021) while some features in omic data such as metabolomics can be explained by a few genetic variants, there is a lot of variability in the genetic architecture of different metabolites and a great deal of uncertainty across omic features (Panyard et al., 2021). Leveraging genome‐wide SNPs to increase the potential predicted heritability for an intermediate is potentially important when extending these types of analyses to a range of omics data in which a large possible number of SNPs may contribute to the variation or heritability in each omic feature (Darst et al., 2019). As in all analyses using IVs, the analysis is constrained by the need for each intermediate to have at least one SNP that explains a sufficient amount of variation to be useful for prediction. Identifying such SNPs may be a limiting step in approaches requiring individual‐level data because often the availability of all the individual level data is limited. However, since SHA‐JAM uses summary statistics to estimate the effects for predicting the intermediate, this creates the opportunity for publicly available data and subsequent meta‐analyses to be leveraged, providing well‐powered summary statistics to identify and estimate SNP effects for the construction of the weight matrix. This agnostic construction of the weight matrix identifies each SNPs while treating each intermediate as independent. This type of selection can potentially introduce SNPs that may be influential for other intermediates. However, compared to MR‐BMA, SHA‐JAM demonstrated robust estimation to potential invalid instrument variables for any specific intermediate (Zuber et al., 2020).

Additionally, the summary data for extracting the genetic variants and intermediates for weight matrix determine the candidate intermediates for selection. One may miss some previously reported intermediates due the lack of SNP‐intermediate pairs when identifying the weight matrix. For example, in our gene‐prostate cancer example, we did not include *HNF1B (*Eeles et al., 2008 *)* due to the lack of significant eQTL‐*HNF1B* pairs existing in GTEx prostate tissue, although this gene has been strongly implicated gene for expression in prostate cancer risk. Such problems may be avoided by conducting a more comprehensive search, such as identifying the eQTL‐gene pairs in all tissues instead of a single tissue.

For the agnostic selection of high‐dimensional omic intermediates, often with potentially correlated features, SHA‐JAM provides credible sets for variable selection via SuSiE. This leverages highly correlated intermediates with small effects to identify common effects and summarizes the uncertainty in selection within the correlated intermediates. In the data example of selecting the metabolites for prostate cancer risk, we identified four metabolites, though with small effects. The findings were consistent with previous large epidemiological studies (Kok et al., 2011; Orho‐Melander et al., 2018). A review by Suburu and Chen (2012) illustrated the strong impact of de novo lipogenesis on prostate cancer. For example, cholesterol that is derived from the mevalonic acid pathway could impact the development and progression of prostate cancer. Statins, which lower cholesterol levels by inhibiting HMG‐CoA reductase, the rate‐limiting enzyme in cholesterol synthesis, showed a protective effect on prostate cancer risk in several large cohort studies (Bansal et al., 2012; Boudreau et al., 2008; Chen et al., 2022). This was consistent with the effect of an *HMGCR* locus, rs7703051, shown in our analysis which was significantly associated with increased cholesterol in small LDL, LDL, and IDL and total serum cholesterol. In the other data example of selecting genes for the risk of prostate cancer, we were able to identify several credible sets which contained highly correlated genes, each with small effects and each with a low posterior inclusion probability. These genes may not be selected in approaches based on the univariate estimates or MIP. Several discovered genes are genes reported in previous GWAS and gene expression studies (Table 4). For example, *ACPP* (Wayner et al., 2012) (PIP = 0.999) was overexpressed in prostate cancer cell lines and *HOTAIRM1* (PIP = 0.999) is antisense of *HOXA1* and found to be highly expressed in prostate cancer cells enhancing cell proliferation, invasion, and metastasis (Wang et al., 2015). Several genes that were not previously reported to prostate cancer but have been observed in association with other cancers or associated with risk factors for prostate cancer were identified by SHA‐JAM. The most noteworthy gene is *L3MBTL3* (PIP = 1) which has not previously been linked to prostate cancer but has been reported to be associated with height (Gudbjartsson et al., 2008), type 2 diabetes (Vujkovic et al., 2020), and body mass index (Pulit et al., 2019), all suspected risk factors of prostate cancer (Table 4).

In the context of both SNP and intermediate selection, another critical benefit of SHA‐JAM is that it shares the computational efficiency of SuSiE (Wang et al., 2020) and runs ~300 times faster than MR‐BMA when 50 candidate intermediates are examined in the model. This computational advantage increases as the number of candidate intermediates or SNPs increases. Another intermediate selection algorithm that we tested in this paper, EN‐hJAM, also showed the computational efficiency but a relatively more conservative selection comparing to SHA‐JAM. Moreover, EN‐hJAM does not provide the evidence of strength which makes it unlikely for one to draw statistical inference (Lee et al., 2016; Taylor & Tibshirani, 2015; Tibshirani et al., 2016).

Despite the benefits from SuSiE and hJAM, SHA‐JAM shares the caveats of both SuSiE and IV analysis, such as decreased performance with unknown or unaccounted for pleiotropy. In addition, SuSiE may not perform well and may not converge in the presence of sparse data with many causal effects (Wang et al., 2020). In our simulation studies, this led to a decrease in performance for SHA‐JAM as the number of causal intermediates increased (Table 2 and Figure 2). Wang et al. (2020) have proposed several possible solutions, such as using a better initialization for the algorithm and this is an area of potential future research. As mentioned previously these types of analyses require assumptions, such as no pleiotropy and linear effects of SNPs on the intermediates, that may not be true in practice. This is an active area of research and novel approaches exist to relax these assumptions such as Tang et al., Variance‐Component TWAS (VC‐TWAS) (Tang et al., 2021) which utilizes the kernel similarity.

For applications with summary data, SuSiE can be very sensitive to the accuracy of the reference genotype LD structure to the data generating the summary statistics (Bhattacharya et al., 2020; Jiang et al., 2021; Wainberg et al., 2019). Genotype reference data that are not representative may cause a large bias in computing the correlation structure of the SNPs and can eventually lead to inaccurate results. At a minimum, in application, the reference genotype data need to share the same ancestry as the populations used to generate the summary statistics. For summary statistics obtained from trans‐ancestry meta‐analysis or from admixed populations, this may not be sufficient (Conti et al., 2021). We are currently developing an extension of SHA‐JAM that explicitly models the population‐specific LD from different groups and then compute a multi‐population average effect.

Another limitation is that SHA‐JAM does not account for the uncertainty in the estimates of the SNP‐intermediates obtained from external data through a fully Bayesian model; thus, the PIP from the model may not represent all existing uncertainty. To the best of our knowledge, it is a shared limitation of existing MR or TWAS approaches. From an uncertainty and measurement error perspective, our approach is most similar to an expectation substitution approach, which can perform well and has advantages of simplicity and flexibility while not propagating all the uncertainty to be reflected in the final estimates (Kraft et al., 2005; Stram et al., 2003). Thus, we consider our use of joint estimates obtained from the first step in SHA‐JAM an adequate tradeoff for gains in straightforward application while provided valid and robust estimates for subsequent downstream analyses. Moreover, we demonstrate with our simulation that the PIPs are sufficiently estimated to be used for inference (Supporting Information S1: Figure 10). As an alternative, we have previously implemented a full hierarchical model linking an experimental omic study to an epidemiologic study (Li et al., 2012). However, it requires access to individual‐level data from both study populations and is computationally intensive even in a small dimensional situation. As high dimensional data are becoming more widely available, we believe a computationally efficient approach that can be flexible in its implementation, such as constructing a valid weight matrix with joint estimates from marginal summary statistics, is of more potential use.

In summary, we introduce the well‐performing SHA‐JAM, a two‐step scalable approach to construct a weight matrix and perform intermediate variable selection using summary statistics from omics data. With SHA‐JAM, we identified 4 out of 30 tested metabolites with small effects and 55 out of 1098 tested genes, including 37 genes that have not been previously reported, that are associated with the prostate cancer risk. SHA‐JAM can detect small effects and select highly correlated intermediates with credible sets and is more computationally efficient than existing approaches.

## CONFLICT OF INTEREST STATEMENT

The authors declare no conflict of interest.

## Supporting information

Supporting information.

## Data Availability

Implementations of SHA‐JAM and EN‐hJAM are available on Github (https://github.com/USCbiostats/hJAM). The sample data for simulations and the two data example are also available on Github (https://github.com/USCbiostats/hJAM/tree/master/data).
