# Supplementary material for: Hierarchical joint analysis of marginal summary statistics—Part II: High‐dimensional instrumental analysis of omics data
Source: Genet Epidemiol. 2024 Jun 17;48(7):291–309. doi: 10.1002/gepi.22577 (PMC12333930; doi:10.1002/gepi.22577)
Supplement: Supplementary file 1 — Supporting information. [file GEPI-48-291-s001.docx]

**SHA-JAM: A Scalable Hierarchical Approach for Joint Analysis with Marginal GWAS Summary Statistics from Omics Data**

#### Supplementary Methods

**Constructing hJAM variables**

We construct the plug-ins of $\boldsymbol{G}^{'}\boldsymbol{G}$, $\boldsymbol{G}'\boldsymbol{x}$ and $\boldsymbol{x}'\boldsymbol{x}$, following previous description of JAM ^1,2^. To avoid an intercept term, all genotype data are centered as $g_{ik}=-2p_{k}, 1-2p_{k}$ or $2-2p_{k}$ where $g_{ik}$ denotes the dosage of $k^{th}$ SNP for individual $i$. We define $\boldsymbol{z}:=\boldsymbol{G'x}$ as in JAM ^1^ and construct $\boldsymbol{z}$ with the genotype group means $\bar{g}_{ks}$ and counts for each group $n_{ks}$, where $s=\{0, 1, 2\}$ the number of alleles for each SNP *k*. Assuming Hardy-Weinberg equilibrium (HWE), we have $\hat{n}_{k0}=\left( 1-\hat{p}_{k} \right)^{2}n$,  $\hat{n}_{k1}=2 \hat{p}_{k}\left( 1-\hat{p}_{k} \right)n$, and  $\hat{n}_{k2}=\hat{p}_{k}^{2}n$, where $\hat{p}_{k}$ denotes the allele frequency of the effect allele and $n$ is the sample size of the summary data. Note that $\hat{p}_{k}$ could be obtained from the reference panel or from data set providing the GWAS for the intermediates. The overall group means for SNP $g_{k}$ can be expressed as

|  | $\bar{g}_{k.}=\frac{\hat{n}_{k0}\bar{g}_{k0}+\hat{n}_{k1}\bar{g}_{k1}+\hat{n}_{k2}\bar{g}_{k2}}{\hat{n}_{k0}+\hat{n}_{k1}+\hat{n}_{k2}}=0$ | (6) |
| --- | --- | --- |

since $\boldsymbol{G}$ is mean centered. Under the assumption of additive effects of alleles, we have  $\bar{g}_{k1}=\bar{g}_{k0}+\hat{\alpha}_{km}$ and $\bar{g}_{k2}=\bar{g}_{k0}+{2\hat{\alpha}}_{km}$. Substituting the two equations into Eq. 6, we approximate the genotype group means of $\bar{g}_{k1}$ and $\bar{g}_{k2}$ giving $z_{k}={\hat{n}_{k1}\bar{g}}_{k1}+2{\hat{n}_{k2}\bar{g}}_{k2}$. Under a linear regression model, the marginal and joint effect estimates of SNPs on intermediate $m$ can be expressed as ${\hat{\boldsymbol{\alpha}}}_{\boldsymbol{m}}\boldsymbol{=}\boldsymbol{D}^{\boldsymbol{-1}}\boldsymbol{G}^{\boldsymbol{'}}\boldsymbol{x}_{\boldsymbol{m}}$ and ${\hat{\boldsymbol{a}}}_{m}=\left( \boldsymbol{G}^{'}\boldsymbol{G} \right)^{-1}\boldsymbol{G}^{\boldsymbol{'}}\boldsymbol{x}_{m}$ with the same variance $\mathrm{var}\left( {\hat{\boldsymbol{a}}}_{\boldsymbol{m}} \right)={\sigma_{e}^{2}\left( \boldsymbol{G}^{\boldsymbol{'}}\boldsymbol{G} \right)}^{-1}$, respectively, where $\boldsymbol{D}$ is the diagonal matrix of $\boldsymbol{G'G}$ with $D_{k}=\sum_{i=1}^{n} g_{ik}^{2}$ and $\sigma_{e}^{2}$ denotes the residual variance in the joint analysis. Since $g_{ik}^{2}$ is not available in summary data, we approximate $D_{k}=2\hat{p}_{k}\left( 1-\hat{p}_{k} \right)n$ assuming HWE. For a single SNP $k$, we have $R_{k, e}^{2}=\frac{\hat{a}_{km}^{'}\boldsymbol{g}_{k}'\boldsymbol{x}}{x'x}=\frac{\hat{a}_{km}^{'}D_{k}\hat{\alpha}_{km}}{x'x}.$Thus, we have $\hat{\sigma}_{k,e}^{2}=\frac{\left( 1-R_{k, e}^{2} \right)x^{'}x}{n-1}=\frac{x^{'}x-D_{k}{\hat{\alpha}^{2}}_{km}}{n-1},$and squared standard error of the estimate is $S_{k}^{2}=\hat{\sigma}_{k,e}^{2}/D_{k}$. Thus, we have $x^{'}x=D_{k}S_{k}^{2}\left( n-1 \right)+D_{k}{\hat{\alpha}^{2}}_{km}$. Following Yang et al. ^2^, we have the plug-in of $x'x$ as the median of $D_{k}S_{k}^{2}\left( n-1 \right)+D_{k}{\hat{\alpha}^{2}}_{km}$ across all SNPs. Denote $\boldsymbol{W}$ as the reference genotype data with a sample size $n_{W}$, we have $\boldsymbol{G}^{\boldsymbol{'}}\boldsymbol{G}=\boldsymbol{W'W}$ when the $n_{W}=n$. When the reference data sample size differs from the GWAS providing the summary statistics, we adjust the variance-covariance matrix (i.e., $\boldsymbol{W'W}$) by defining $D_{w}=\sum_{i=1}^{n_{W}} w_{ik}^{2}$ where $w_{ik}$ denotes the dosage of $k^{th}$ SNP for individual $i$ in $\boldsymbol{W}$, and $\boldsymbol{G}^{\boldsymbol{'}}\boldsymbol{G}$ by

$$G^{'}G_{k_{1},k_{2}}=\sqrt{\frac{D_{k_{1}}D_{k_{2}}}{D_{W, k_{1}}D_{w, k_{2}}}}\sum_{i=1}^{n_{W}} w_{i, k_{1}}w_{i, k_{2}}.$$

#### Elastic net hJAM

In our previous work hJAM ^3^, we provided a framework to perform the Mendelian randomization or TWAS with a few intermediates and at most moderately correlated SNPs. As we described in the methods section, the instrumental variable analysis could be expressed at a two-stage model when individual level data is available. Stage 1 models the outcome as a linear function of the genetic variants,

$$\boldsymbol{y}=\boldsymbol{G\beta}+\boldsymbol{e},$$

and stage 2 models the effect estimates $\beta$ as a function of the weight matrix,  $\hat{A}$,

$$\boldsymbol{\beta}=\hat{\boldsymbol{A}}\boldsymbol{\pi}+\boldsymbol{\epsilon}.$$

Here $\boldsymbol{y}$ is a $n$-length vector of outcome, $\boldsymbol{G}$ is the genotype data, and $\hat{\boldsymbol{A}}\mathbb{\in R}^{K\times M}$ is the weight matrix which is composed by the pairwise association estimates between $K$ SNPs and $M$ intermediates. Similar to the section “constructing hJAM variable” as above, we first computed the total trait burden $\boldsymbol{z}:=\boldsymbol{G}_{\boldsymbol{0}}^{\boldsymbol{'}}\boldsymbol{y}$ for the risk alleles of SNPs using the marginal summary statistics,  $\hat{\boldsymbol{b}}$, the effect estimates of $\boldsymbol{\beta}$, the minor allele frequency $\hat{p}$ of each genetic variant, and $N_{y}$, the total sample size of the genome-wide association studies (GWAS) of the outcome. With standard linear algebra, we then express the total trait burden as a multivariate normal distribution as

$$\boldsymbol{z}\sim MVN_{K}\left( \boldsymbol{G}_{\mathbf{0}}^{'}\boldsymbol{G}_{\mathbf{0}} \boldsymbol{\beta}, \sigma_{y}^{2}\boldsymbol{G}_{\mathbf{0}}^{'}\boldsymbol{G}_{\mathbf{0}} \right)$$

where $\boldsymbol{G}_{\mathbf{0}}^{'}\boldsymbol{G}_{\mathbf{0}}$ is a $K\times K$ genotyped variance-covariance matrix from an external centered-by-mean genotype data. Plug in the stage 2 model, we have

$$\boldsymbol{z}\sim MVN_{K}\left( \boldsymbol{G}_{\mathbf{0}}^{'}\boldsymbol{G}_{\mathbf{0}}\hat{\boldsymbol{A}}\boldsymbol{\pi}, \sigma_{y}^{2}\boldsymbol{G}_{\mathbf{0}}^{'}\boldsymbol{G}_{\mathbf{0}} \right).$$

To simplify the likelihood, Cholesky decomposition transformation $\boldsymbol{L}^{'}\boldsymbol{L}=\boldsymbol{G}_{\mathbf{0}}^{'}\boldsymbol{G}_{\mathbf{0}}$ is performed and $\boldsymbol{z}$ vector is transformed into $\boldsymbol{z}_{\boldsymbol{L}}$ with the inverse of $\boldsymbol{L}'$:

|  | $\boldsymbol{z}_{\boldsymbol{L}}\sim MVN_{K}\left( \boldsymbol{L}\hat{\boldsymbol{A}}\boldsymbol{\pi}, \sigma_{y}^{2} \boldsymbol{I}_{\boldsymbol{K}} \right).$ | (S1) |
| --- | --- | --- |

Benefit from the independent correlation structure of dependent intermediates in Eq. S1, we can perform regularization to variable selection in regression. The generalized objective function is

$$\hat{\boldsymbol{\pi}}=\underset{\boldsymbol{\pi}}{\mathrm{argmin}} \left( \left\| \boldsymbol{z}_{\boldsymbol{L}}-\boldsymbol{L}\hat{\boldsymbol{A}}\boldsymbol{\pi} \right\|^{2}+\lambda_{1}\left\| \boldsymbol{\pi} \right\|_{2}^{2}+\lambda_{2}\left\| \boldsymbol{\pi} \right\|_{1} \right),$$

where $\left\| \boldsymbol{\pi} \right\|_{1}=\sum_{m=1}^{M} |\pi_{m}|$ and $\left\| \boldsymbol{\pi} \right\|^{2}=\sum_{m=1}^{M} \pi_{m}^{2}$. Denote $c:=\frac{\lambda_{2}}{\lambda_{1}+\lambda_{2}}$ and $\lambda=\lambda_{1}+\lambda_{2}$, we can express the regularized hJAM problem as

$$\hat{\boldsymbol{\pi}}=\underset{\boldsymbol{\pi}}{\mathrm{argmin}} \left( \left\| \boldsymbol{z}_{\boldsymbol{L}}-\boldsymbol{L}\hat{\boldsymbol{A}}\boldsymbol{\pi} \right\|^{2}+\lambda\left( 1-c \right) \left\| \boldsymbol{\pi} \right\|_{2}^{2}+\lambda c\left\| \boldsymbol{\pi} \right\|_{1} \right),$$

When $c=0$, we have a ridge hJAM; and when $c=1$, we have a LASSO hJAM. We use the same optimization algorithms to solve the regularzed hJAM problem as in the original elastic net paper ^4^.

##### Fitting hJAM for Selection of Intermediates

Given a weight matrix, we apply the Sum of Single Effect regression (SuSiE) ^31^ to select the intermediates in the hJAM framework (Eq. 3). SuSiE is a recently proposed approach for variable selection for highly correlated data with sparse detectable effects such as in fine mapping for GWAS ^31^, and offers computational efficient selection that benefit from a iterative Bayesian Stepwise selection (IBSS) algorithm, which is analogous to a forward selection. As its name suggests, the SuSiE model for an outcome is constructed as the sum of multiple Single Effect Regression (SER) models. SER assumes that out of the $M$ variables in Eq. 3, there exists exactly one non-zero regression coefficient:

$$\boldsymbol{\pi}=\pi\boldsymbol{\gamma, \gamma}\sim\mathrm{Mult}\left( 1, \tau\right) \mathrm{and} \pi\sim N\left( 0, \sigma_{0}^{2} \right),$$

where $\boldsymbol{\gamma\in}\left\{ 0, 1 \right\}^{M}$ is a $M$-length vector of indicator variables that gives the prior probability for each variable being the non-zero one and $\pi$ is a scalar for the “single effect”. Here, $\sigma_{0}^{2}$ denote the prior variance of the non-zero effect. Given individual-level data ($\boldsymbol{G}$ and $\boldsymbol{y}$), the weight matrix ($\hat{\boldsymbol{A}}$), the pre-set residuals of the outcome in Eq. 1 ($\sigma^{2}$), and $\sigma_{0}^{2}$, we can obtain the posterior distribution as described in Wang et al. ^31^:

$\boldsymbol{\gamma|}\hat{\boldsymbol{X}}, \boldsymbol{y}, \sigma^{2},\sigma_{0}^{2}\sim\mathrm{Mult}\left( 1, \boldsymbol{\nu} \right),$ where $\hat{\boldsymbol{X}}\boldsymbol{:=G}\hat{\boldsymbol{A}}$

$$\pi| \hat{\boldsymbol{X}},\boldsymbol{y}, \sigma^{2}, \sigma_{0}^{2}, \gamma_{m}=1\sim N\left( \mu_{1m}, \sigma_{1m}^{2} \right).$$

Here, $\boldsymbol{\nu}$ is a $M$-length vector of posterior inclusion probabilities (PIPs) under the SER model and $\nu_{m}\boldsymbol{:=}Pr(\gamma_{m}\boldsymbol{=}1\boldsymbol{|}\hat{\boldsymbol{X}}, \boldsymbol{y}, \sigma^{2},\sigma_{0}^{2}\boldsymbol{)}$for the $m^{th}$ intermediate. $\mu_{1m}$ and $\sigma_{1m}^{2}$ are the posterior mean and variance of $\pi$ given $\gamma_{m}=1$, which can be computed as a Bayesian simple linear regression. Thus, each SER gives a single distribution for $\pi$ and $\boldsymbol{\gamma}$.

The SuSiE model for variable effects is a mixture of $L$ SER effects, expressed as

|  | $\boldsymbol{\pi}=\sum_{l=1}^{L} \boldsymbol{\pi}_{\boldsymbol{l}}= \sum_{l=1}^{L} \pi_{l} \boldsymbol{\gamma}_{\boldsymbol{l}}$ | (4) |
| --- | --- | --- |
|  | $\boldsymbol{\gamma}_{\boldsymbol{l}}\sim\mathrm{Mult}\left( 1, \tau\right) \mathrm{and} \pi_{l}\sim N\left( 0, \sigma_{0l}^{2} \right),$ | (5) |

where $l=1, \ldots, L$, where *L* denotes the maximum number of credible sets allowed and is pre-specified and $\sigma_{0l}^{2}$ is the variance of the prior for $\pi$ in the $l^{th}$ credible set. Here SuSiE assigns a univariate variance, $\sigma_{0l}^{2}$, to each $\pi_{l}$ across $L$ credible sets. The set of $\sigma_{0l}^{2}$ and $\sigma^{2}$ in Eq. 1 could be updated using expectation-maximization algorithm, as discussed in the SuSiE paper ^31^. When $L=1$, SuSiE returns a single SER model.

To fit hJAM with SuSiE framework, we adopted the IBSS algorithm ^31^. At each iteration, given current estimates of $\boldsymbol{\pi}_{\boldsymbol{l'}}$**,** we compute the expected residual without the single effect, $\pi_{l}$ ($l\neq l^{'})$, and update the estimate of $\boldsymbol{\pi}_{\boldsymbol{l}}$ with the expected residual fitting a SER model. Details are described in Algorithm 1 and 4 of Wang et al. ^31^. Note that for each dependent variable $m$, $\pi_{lm}$ is independent across $l$ because of the property of the variational approximation algorithm that SuSiE employs in computing the posterior distribution in IBSS ^31^. Therefore, under SuSiE, the posterior mean and posterior inclusion probability (PIP) of the $m^{th}$ variable are

$$\pi^{(m)}:= \sum_{l=1}^{L} \pi_{lm}=\sum_{l=1}^{L} \mu_{lm} \nu_{lm},$$

and

$$\mathrm{PI}P_{m}:=\Pr\left( \pi^{\left( m \right)}\neq0 | \hat{\boldsymbol{X}}, \boldsymbol{y} \right)\approx1-\prod_{l\in L} \left( 1-\nu_{lm} \right),$$

respectively. We then identify the intermediates by computing the ρ-levelcredible sets of intermediates calculated using the PIP for each intermediate and the pre-set minimum absolute correlation structure between the intermediates within each credible set. A ρ-levelcredible set is defined as a subset of intermediates among which at least one intermediate has non-zero effect and the sum of PIPs for these included intermediates equals or greater than $\rho$. The default $\rho$ was set to be 0.95.

**Details in calculating the summary statistics in simulation studies**

To generate the summary statistics that we used in the simulation studies, we first simulate the individual level data as we described in the main text and then calculated the summary statistics from the individual level data. Here we describe the steps in details with the order from the main text.

1. An effect estimates vector $\hat{\boldsymbol{b}}$ and the corresponding $se(\hat{\boldsymbol{b}})$ from $\boldsymbol{G}_{\boldsymbol{y}}$ and $\boldsymbol{y}$: we used univariate linear regression where we regressed each genetic variant on the outcome to obtain the marginal effect estimates and the corresponding standard errors.
2. A vector of the minor allele frequency (MAF) of the SNPs from $\boldsymbol{G}_{\boldsymbol{y}}$ and $\boldsymbol{y}$: we calculated the MAF for each genetic variant in the $\boldsymbol{G}_{\boldsymbol{y}}$, assuming Hardy-Weinberg Equilibrium.
3. A weight matrix $\hat{A}$ matrix from $\boldsymbol{G}_{\boldsymbol{X}}$ and $\boldsymbol{X}$: to construct the $\hat{A}$, we first get the marginal effect estimates for each SNP on each intermediate and then transformed the vector of the marginal effect estimate for each intermediate into different types based on the methods we used for the inference. With SHA-JAM and EN-hJAM, for the vector of each intermediate, we used SuSiE JAM to select the genetic variant and use the posterior inclusion probability (PIP) as the conditional estimates that we use in the $\hat{A}$ matrix. We included the genetic variants which have been selected by SuSiE JAM for at least one intermediate. The implementation is included in <https://github.com/USCbiostats/hJAM/blob/master/R/susieJAM.R>. With MR-BMA, for the vector of each intermediate, we first excluded the univariately non-significant genetic variants and applied priority pruner to prune the genetic variants using a cutoff $R=0.4$ where $R$ is the pairwise correlation between genetic variants. The selected univariate estimates were then transformed into the inverse-variance weighted estimates by the MR-BMA implementation (<https://github.com/verena-zuber/demo_AMD>).
4. A linkage disequilibrium (LD) structure from $\boldsymbol{G}_{\boldsymbol{R}}$: we calculated the LD structure from the $\boldsymbol{G}_{\boldsymbol{R}}$ individual level data.

#### Supplementary Tables

Supplementary Table 1 Averaged absolute bias for the estimates from SHA-JAM, EN-hJAM and MR-BMA across 600 replications.

| $\boldsymbol{r}_{\mathbf{within block}}$**^*^** | $\max\boldsymbol{r}_{\boldsymbol{X}}$**^^^** | **Selection algorithm** | **Number of causal intermediates** | | | |
| --- | --- | --- | --- | --- | --- | --- |
|  |  |  | **0** | **3** | **7** | **10** |
| 0 | 0 | SHA-JAM | 0.002 | 0.003 | 0.011 | 0.019 |
|  |  | EN-hJAM | 0 | 0.011 | 0.024 | 0.034 |
|  |  | MR-BMA | 0.001 | 0.049 | 0.104 | 0.141 |
| 0 | 0.6 | SHA-JAM | 0.001 | 0.003 | 0.012 | 0.021 |
|  |  | EN-hJAM | 0 | 0.011 | 0.025 | 0.033 |
|  |  | MR-BMA | 0.001 | 0.051 | 0.104 | 0.138 |
| 0.6 | 0 | SHA-JAM | 0.001 | 0.005 | 0.018 | 0.031 |
|  |  | EN-hJAM | 0 | 0.016 | 0.036 | 0.050 |
|  |  | MR-BMA | 0.007 | 0.112 | 0.160 | 0.186 |
| 0.6 | 0.6 | SHA-JAM | 0.002 | 0.005 | 0.018 | 0.033 |
|  |  | EN-hJAM | 0 | 0.016 | 0.037 | 0.052 |
|  |  | MR-BMA | 0.007 | 0.112 | 0.161 | 0.185 |
| 0.8 | 0 | SHA-JAM | 0.002 | 0.006 | 0.023 | 0.040 |
|  |  | EN-hJAM | 0 | 0.017 | 0.038 | 0.055 |
|  |  | MR-BMA | 0.007 | 0.095 | 0.134 | 0.152 |
| 0.8 | 0.6 | SHA-JAM | 0.001 | 0.006 | 0.024 | 0.042 |
|  |  | EN-hJAM | 0 | 0.017 | 0.038 | 0.055 |
|  |  | MR-BMA | 0.007 | 0.093 | 0.133 | 0.149 |

Note: ^*^Correlation within block of $\boldsymbol{G}$’s; ^^^Maximum correlation coefficient between intermediates $\boldsymbol{X}$. We showed the absolute bias of each selection algorithm with best performed $\hat{\boldsymbol{A}}$ matrix with the lowest being bolded for each scenario. We used pruned inverse-variance weighted marginal $\hat{\boldsymbol{A}}$ for MR-BMA, SuSiE JAM $\hat{\boldsymbol{A}}$ for SHA-JAM and EN- hJAM.

Supplementary Table 2 Averaged specificity, sensitivity, and positive predictive value (PPV) for 95% credible sets for selection of intermediates for SHA-JAM, EN-hJAM and MR-BMA across all simulation scenarios with differing number of causal intermediates.

| **Metrics** | **Selection algorithm** | **Number of causal intermediates** | | |
| --- | --- | --- | --- | --- |
|  |  | **3** | **7** | **10** |
| Specificity | SHA-JAM | 0.868 | 0.763 | 0.688 |
|  | EN-hJAM | 0.480 | 0.537 | 0.538 |
|  | MR-BMA | 0.745 | 0.595 | 0.537 |
| Sensitivity | SHA-JAM | 0.998 | 0.992 | 0.987 |
|  | EN-hJAM | 0.996 | 0.983 | 0.969 |
|  | MR-BMA | 0.947 | 0.933 | 0.931 |
| PPV | SHA-JAM | 0.992 | 0.963 | 0.928 |
|  | EN-hJAM | 0.969 | 0.932 | 0.900 |
|  | MR-BMA | 0.983 | 0.935 | 0.892 |

Note: The true positives are defined as the true non-zero intermediate being selected by the model: included in any 95% credible sets for SHA-JAM, non-zero effect for EN-hJAM, and a marginal probability larger than 0.2 for MR-BMA.

Supplementary Table 3 Averaged specificity, sensitivity, and positive predictive value (PPV) for 90% credible sets for selection of intermediates for SHA-JAM across all simulation scenarios with differing number of causal intermediates.

| **Metrics** | **Selection algorithm** | **Number of causal intermediates** | | |
| --- | --- | --- | --- | --- |
|  |  | **3** | **7** | **10** |
| Specificity | SHA-JAM | 0.717 | 0.583 | 0.488 |
| Sensitivity | SHA-JAM | 0.991 | 0.983 | 0.976 |
| PPV | SHA-JAM | 0.982 | 0.936 | 0.885 |

Note: The true positives are defined as the true non-zero intermediate being selected by the model: included in any 90% credible sets for SHA-JAM.

Supplementary Table 4 Sensitivity analysis: mean-squared errors and bias of the estimates in different 𝑨 matrix construction, averaged across 500 replicates.

| $\boldsymbol{r}_{\mathbf{within block}}$**^*^** | **Algorithms** | | | |
| --- | --- | --- | --- | --- |
|  | **SuSiE JAM** | **SuSiE IPD (n=5000^)** | **SuSiE IPD (n=500^)** | **Elastic net** |
| Mean-squared error | | | | |
| 0 | 1.02 | 1.00 | 1.05 | 1.09 |
| 0.6 | 1.01 | 1.00 | 1.05 | 1.09 |
| 0.8 | 1.01 | 1.00 | 1.04 | 1.09 |
| Bias | | | | |
| 0 | 0.81 | 0.80 | 0.82 | 0.83 |
| 0.6 | 0.80 | 0.80 | 0.82 | 0.83 |
| 0.8 | 0.80 | 0.80 | 0.82 | 0.83 |

Note: *Correlation within block of $\boldsymbol{G}$’s. ^Displays the sample size of the data to obtain the $\hat{A}$ matrix.

Supplementary Table 5 Computation time: Average runtime (seconds) for SHA-JAM, EN-hJAM and MR-BMA.

| $\boldsymbol{r}_{\mathbf{within block}}$**^*^** | **Selection algorithm** | **Number of causal intermediates** | | |
| --- | --- | --- | --- | --- |
|  |  | **3** | **7** | **10** |
| 0 | SHA-JAM | 0.128 | 0.143 | 0.150 |
|  | EN-hJAM | 0.263 | 0.246 | 0.252 |
|  | MR-BMA | 34.545 | 55.429 | 71.585 |
| 0.6 | SHA-JAM | 0.226 | 0.289 | 0.321 |
|  | EN-hJAM | 0.481 | 0.426 | 0.412 |
|  | MR-BMA | 86.178 | 112.371 | 124.852 |
| 0.8 | SHA-JAM | 0.163 | 0.208 | 0.250 |
|  | EN-hJAM | 0.323 | 0.317 | 0.316 |
|  | MR-BMA | 77.331 | 91.033 | 94.475 |

Note: ^*^Correlation within block of $\boldsymbol{G}$’s. We showed an average runtime across different $\hat{\boldsymbol{A}}$ matrices and different correlation structures between the intermediates since the runtime did not differ within these groups. Runtime for MR-BMA with 500 iterations for the shotgun stochastic search. Runtimes are average across 600 replicates.

Supplementary Table 6 Description of the candidate metabolites tested for causal associations with risk of prostate cancer.

| **Abbreviation** | **Name** | **Sample size** | **Heritability** |
| --- | --- | --- | --- |
| ApoA1 | ApoA1 | 20687 | 5.01% |
| ApoB | ApoB | 20690 | 8.61% |
| Est.C | Esterified cholesterol | 13497 | 7.58% |
| HDL.C | Total cholesterol in HDL | 21555 | 6.12% |
| HDL.D | HDL diameter | 19273 | 10.32% |
| IDL.C | Total cholesterol in IDL | 19273 | 10.89% |
| IDL.TG | Triglycerides in IDL | 19273 | 9.94% |
| L.HDL.C | Total cholesterol in large HDL | 21558 | 8.23% |
| L.VLDL.C | Total cholesterol in large VLDL | 21235 | 3.24% |
| L.VLDL.TG | Triglycerides in large VLDL | 21239 | 2.59% |
| LDL.C | Total cholesterol in LDL | 21559 | 10.72% |
| LDL.D | LDL diameter | 19273 | 3.12% |
| M.HDL.C | Total cholesterol in medium HDL | 21558 | 2.38% |
| M.VLDL.C | Total cholesterol in medium VLDL | 21551 | 5.12% |
| M.VLDL.TG | Triglycerides in medium VLDL | 21241 | 3.19% |
| S.HDL.TG | Triglycerides in small HDL | 21558 | 4.24% |
| S.LDL.C | Total cholesterol in small LDL | 21556 | 8.96% |
| S.VLDL.C | Total cholesterol in small VLDL | 21557 | 6.72% |
| S.VLDL.TG | Triglycerides in small VLDL | 21558 | 4.91% |
| Serum.C | Serum total cholesterol | 21491 | 8.67% |
| Serum.TG | Serum total triglycerides | 21545 | 4.33% |
| SM | Sphingomyelins | 13476 | 3.92% |
| Tot.FA | Total fatty acids | 13505 | 3.35% |
| TotPG | Total phosphoglycerides | 13519 | 4.24% |
| VLDL.D | VLDL diameter | 19273 | 4.65% |
| XL.HDL.C | Total cholesterol in very large HDL | 21540 | 5.05% |
| XL.HDL.TG | Triglycerides in very large HDL | 21536 | 10.76% |
| XL.VLDL.TG | Triglycerides in very large VLDL | 21548 | 2.41% |
| XS.VLDL.TG | Triglycerides in very small VLDL | 19273 | 7.13% |
| XXL.VLDL.TG | Triglycerides in chylomicrons and extremely large VLDL | 21540 | 1.71% |

Note: This table was adapted from the “NMRA_dat” data set in the MRChallenge R package (<https://github.com/WSpiller/MRChallenge2019>). Four metabolites highlighted in dark grey were excluded in the analysis without the influential variants (K=140).

Supplementary Table 7 Top “best” models from MR-BMA with the influential-variants-excluded ($\boldsymbol{K=140}$) and for estimating the causal effect of metabolites on the risk of prostate cancer.

|  | **Metabolites combination** | **Posterior probability** | **Model specific Causal estimates** |
| --- | --- | --- | --- |
| 1 | Serum.C | 0.059 | 0.063 |
| 2 | S.LDL.C | 0.048 | 0.056 |
| 3 | IDL.C | 0.046 | 0.054 |
| 4 | LDL.C | 0.041 | 0.05 |
| 5 | S.VLDL.C | 0.035 | 0.044 |
| 6 | XXL.VLDL.TG | 0.031 | 0 |
| 7 | ApoA1 | 0.03 | 0.035 |
| 8 | XL.VLDL.TG | 0.029 | 0.006 |
| 9 | ApoB | 0.029 | 0.036 |
| 10 | L.VLDL.TG | 0.028 | 0 |

Abbreviations: Serum.C, serum total cholesterol; S.LDL.C, total cholesterol in small LDL; IDL.C, total cholesterol in IDL; LDL.C, total cholesterol in LDL; S.VLDL.C, total cholesterol in small VLDL; XXL.VLDL.TG, triglycerides in chylomicrons and extremely large VLDL; XL.VLDL.TG, triglycerides in very large LDL; L.VLDL.TG, triglycerides in large VLDL.

Supplementary Table 8 The weight matrix $\hat{\boldsymbol{A}}$ for each chromosome and the corresponding credible sets identified by SuSiE hJAM in selecting the causal gene for the risk of prostate cancer.

| **Chromosome** | **Candidate eQTLs (**$\boldsymbol{K}$**)** | **Candidate genes (**$\boldsymbol{M}$**)** | **Number of CSs** | **Absolute correlation** | | | **Selected genes** |
| --- | --- | --- | --- | --- | --- | --- | --- |
|  |  |  |  | **Minimum** | **Mean** | **Median** |  |
| 1 | 792 | 108 | 1 | 1 | 1 | 1 | 1 |
| 2 | 756 | 78 | 5 | 1 | 1 | 1 | 5 |
| 3 | 469 | 66 | 6 | 0.987 | 0.993 | 0.993 | 7 |
| 4 | 366 | 40 | 0 | - | - | - | 0 |
| 5 | 453 | 57 | 4 | 0.998 | 0.999 | 0.999 | 6 |
| 6 | 1162 | 53 | 5 | 1 | 1 | 1 | 5 |
| 7 | 695 | 65 | 3 | 1 | 1 | 1 | 3 |
| 8 | 351 | 35 | 1 | 0.777 | 0.888 | 0.888 | 2 |
| 9 | 372 | 47 | 2 | 1 | 1 | 1 | 6 |
| 10 | 447 | 52 | 4 | 1 | 1 | 1 | 4 |
| 11 | 470 | 44 | 3 | 1 | 1 | 1 | 3 |
| 12 | 354 | 47 | 2 | 1 | 1 | 1 | 2 |
| 13 | 99 | 9 | 1 | 0.976 | 0.988 | 0.988 | 2 |
| 14 | 246 | 22 | 1 | 0.924 | 0.962 | 0.962 | 2 |
| 15 | 349 | 29 | 0 | - | - | - | 0 |
| 16 | 585 | 48 | 0 | - | - | - | 0 |
| 17 | 512 | 66 | 3 | 1 | 1 | 1 | 3 |
| 18 | 144 | 17 | 1 | 1 | 1 | 1 | 1 |
| 19 | 659 | 65 | 1 | 1 | 1 | 1 | 1 |
| 20 | 189 | 26 | 1 | 1 | 1 | 1 | 1 |
| 21 | 121 | 12 | 0 | - | - | - | 0 |
| 22 | 302 | 42 | 1 | 1 | 1 | 1 | 1 |
| **Total** | **9893** | **1028** | **45** | **0.991** | **0.995** | **0.995** | **55** |

Abbreviations: CSs, Credible sets

Supplementary Table 9 Credible sets that contain two genes, identified by SHA-JAM for selecting the causal genes for the risk of prostate cancer.

| **Chromo-some** | **Gene Name** | **Gene Type** | **Posterior mean** | **PIP** | $\boldsymbol{r}$**^** | **Ref.** |
| --- | --- | --- | --- | --- | --- | --- |
| **Credible sets that contain two genes** | | | | | |  |
| 3 | AMT | *protein coding* | -0.109 | 0.798 | 0.920 |  |
|  | NICN1 | *protein coding* | -0.026 | 0.171 |  |  |
| 5 | CTD-2194D22.3 | *antisense* | -0.226 | 0.885 | 0.994 | ^5^ |
|  | IRX4 | *protein coding* | -0.024 | 0.116 |  | ^5,6^ |
| 5 | CTD-2280E9.1 | *antisense* | -0.030 | 0.574 | 0.997 |  |
|  | CTC-210G5.1 | *antisense* | -0.037 | 0.409 |  |  |
| 8 | FAM66A | *processed transcript* | 0.041 | 0.949 | 0.777 |  |
|  | RP11-351I21.6 | *pseudogene* | 0.000 | 0.012 |  |  |
| 13 | RP11-173B14.4 | *sense intronic* | 0.056 | 0.784 | 0.976 |  |
|  | RP11-173B14.5 | *antisense* | 0.006 | 0.189 |  |  |
| 14 | HAUS4 | *protein coding* | 0.026 | 0.501 | 0.924 |  |
|  | RBM23 | *protein coding* | -0.020 | 0.484 |  |  |
| **Credible sets that contain five genes** | | | | | |  |
| 9 | RP11-203I2.1 | *pseudogene* | -0.261 | 0.201 | 1 |  |
|  | RP11-282E4.1 | *pseudogene* | -0.094 | 0.201 |  |  |
|  | MYO5BP2 | *pseudogene* | -0.020 | 0.201 |  |  |
|  | RP11-262H14.3 | *lincRNA* | -0.021 | 0.201 |  |  |
|  | RP11-262H14.7 | *pseudogene* | -0.021 | 0.201 |  |  |

Note: ^$r$: the correlation coefficient between the genes in the credible set.

Abbreviations: PIP, posterior inclusion probability. Ref., reference.

#### Supplementary Figures


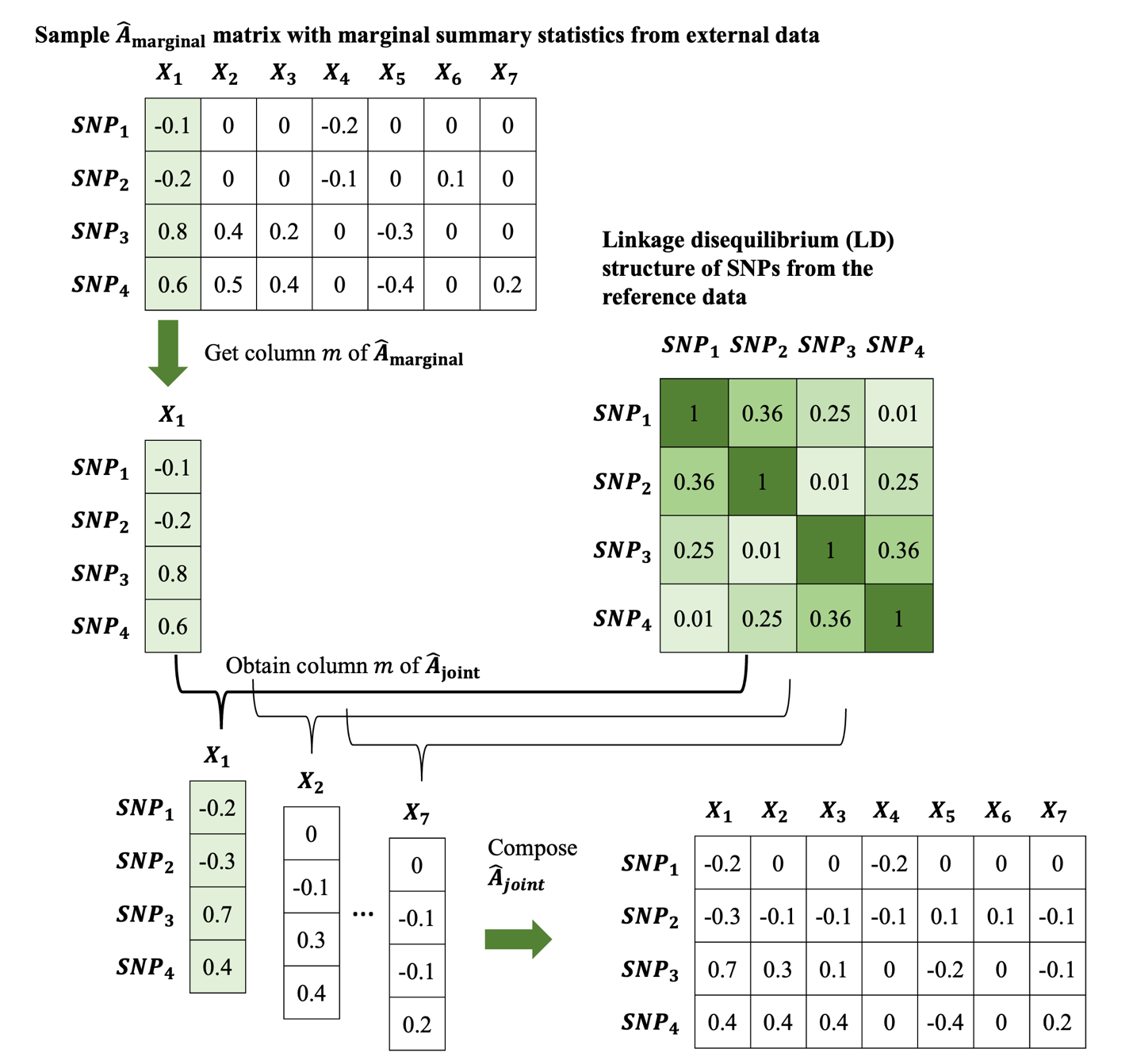


Supplementary Figure 1 Schematic for composing the weight matrix, $\hat{\boldsymbol{A}}$.

This Figure describes the schematic for composing the weight matrix,  $\hat{A}$. In general,  $\hat{A}$ refers to the weight matrix that is composed by the association estimates between the intermediates and the genetic variants. Here, we deliberately described it as  $\hat{A}_{marginal}$ and  $\hat{A}_{joint}$ to specify the different elements (i.e., marginal and joint, respectively) in the weight matrix.


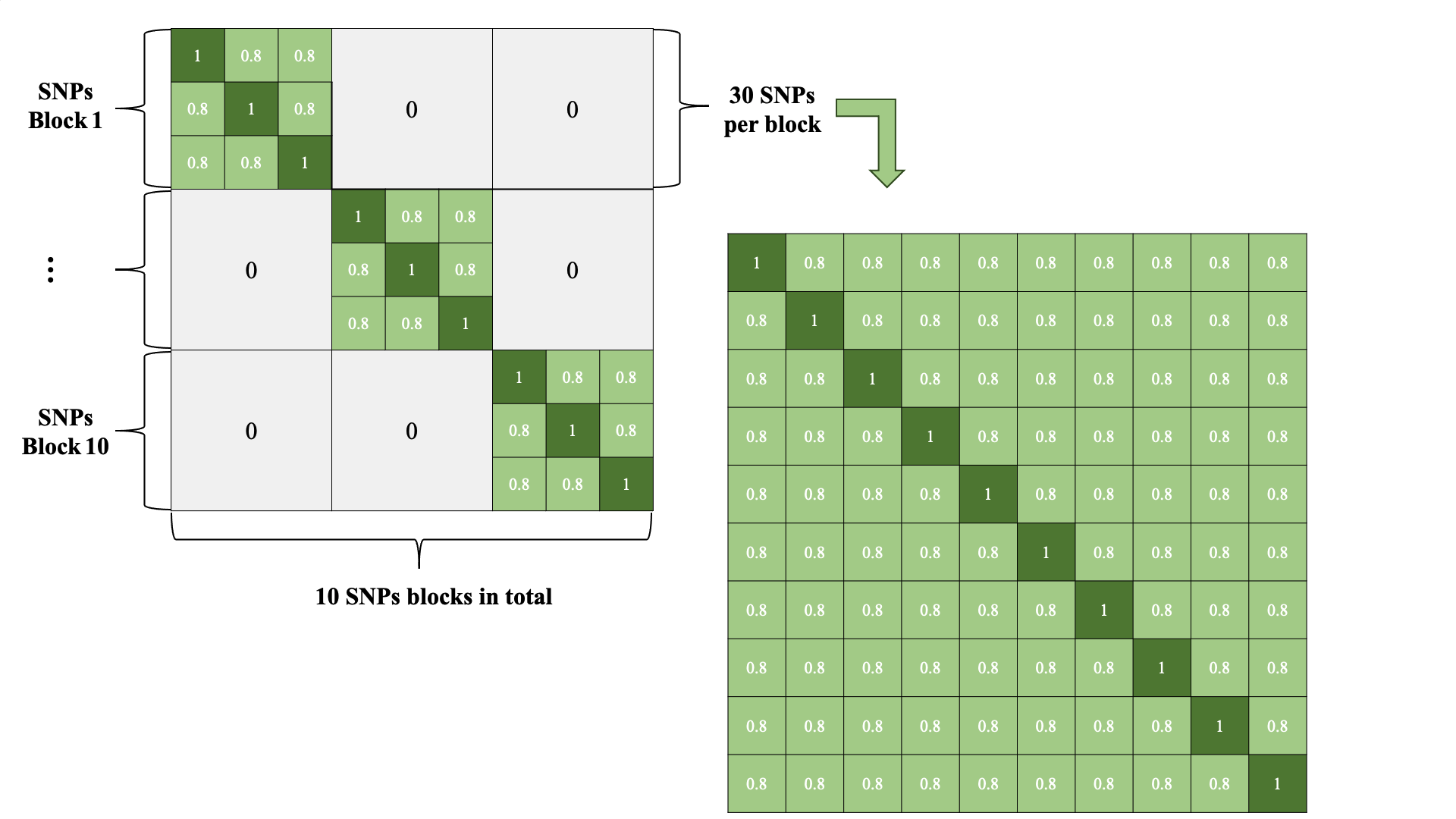


Supplementary Figure 2 Linkage disequilibrium (LD) structure for the simulated individual genotype data set with $\boldsymbol{r}_{\mathbf{within block}}\boldsymbol{=0.8.}$

This Figure displays the LD structure of the individual genotype data with 300 single-nucleotide polymorphism (SNPs) and 10 independent LD blocks. Within each block, the pairwise correlation coefficient between SNPs is $r_{within block}=0.8$. The plot on left shows a large-scale overall LD structure while the plot on right shows details of the true LD structure within one block for the simulation.


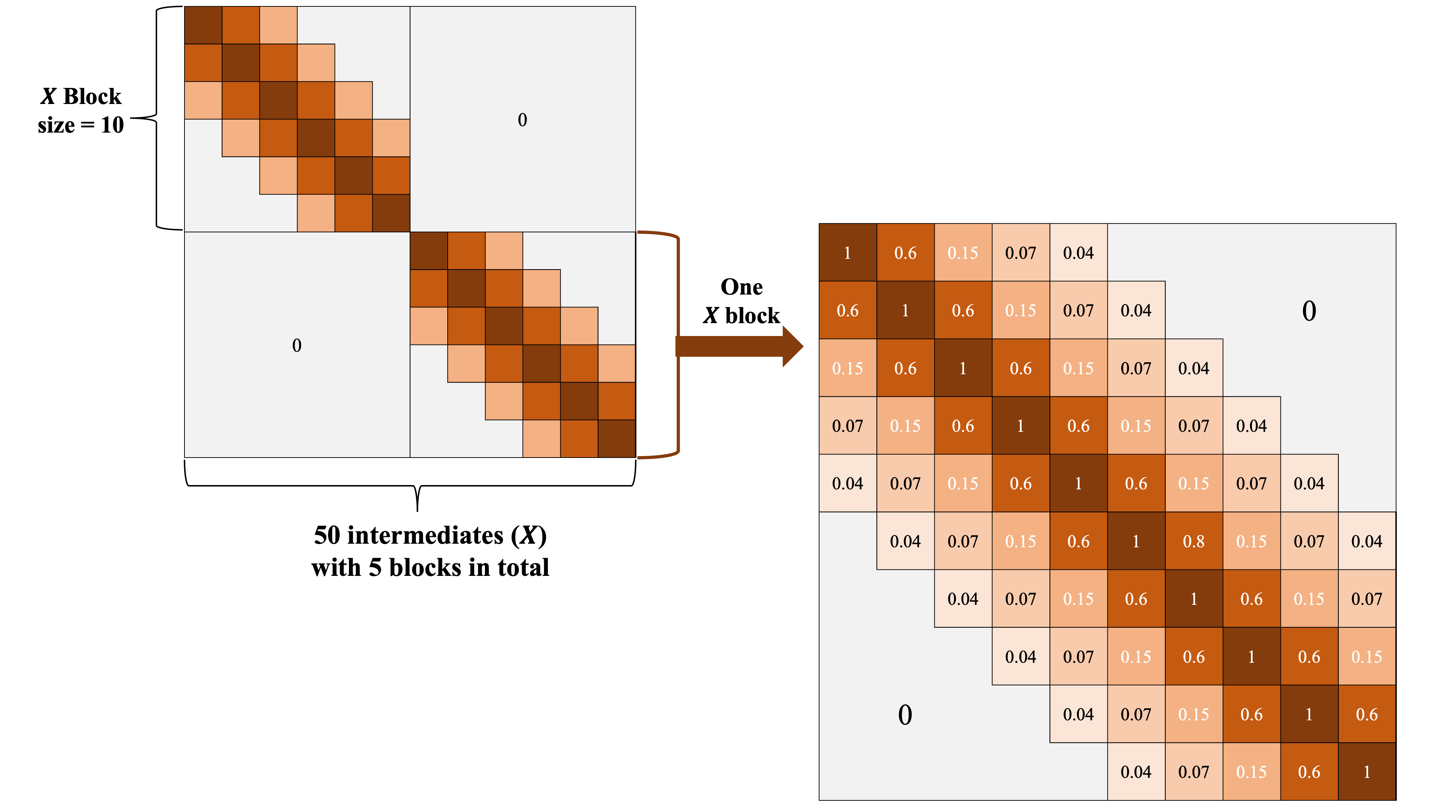


Supplementary Figure 3 Correlation structure for the individual intermediates with ${\text{max}\boldsymbol{(r}}_{\boldsymbol{X}}\boldsymbol{)=0.6}$.

This Figure displays the correlation structure of the individual intermediates data with 50 intermediates (i.e. $\boldsymbol{X}$). The block size of intermediates is 10. For each intermediate $x$, we set 4-8 correlated intermediates with ${\text{max}(r}_{X})=0.6$ and $\min\left( r_{X} \right)=0.04$. The plot on the left shows a large-scale overall correlation structure while the plot on the right shows details of the true correlation structure for one $X$ block for the simulation.


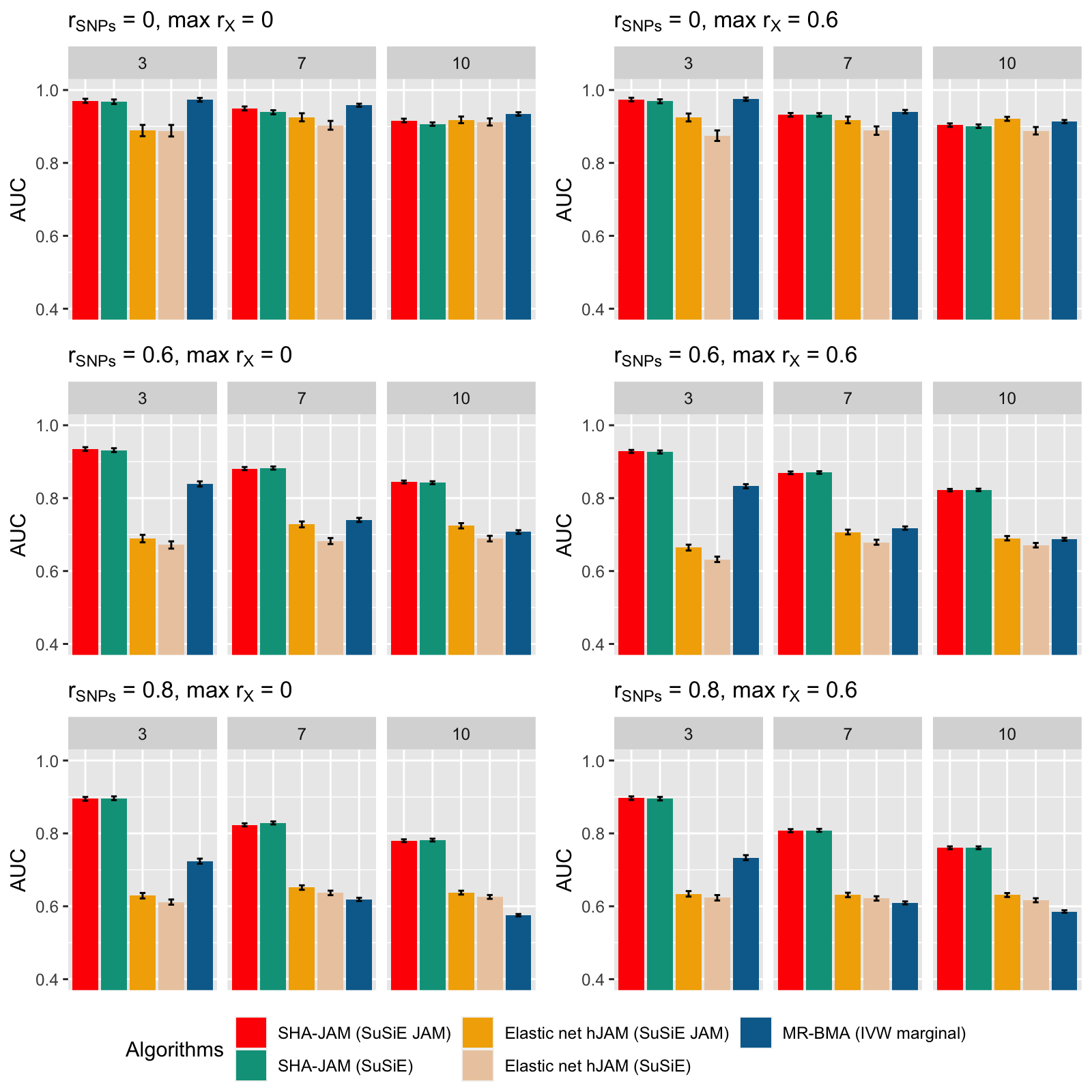


Supplementary Figure 4 Intermediate selection: AUC comparison for SHA-JAM, EN- hJAM and MR-BMA with different weight matrices

Simulation results for three LD structures with different number of causal intermediates. The bar plots showed the average AUC values, and the error bars showed the corresponding standard error. The algorithms are displayed as “Selection algorithm (weight matrix algorithm)”. All weight matrices were composed from data with a sample size of 5,000. SuSiE JAM and IVW marginal used the summary data and SuSiE used the individual data.


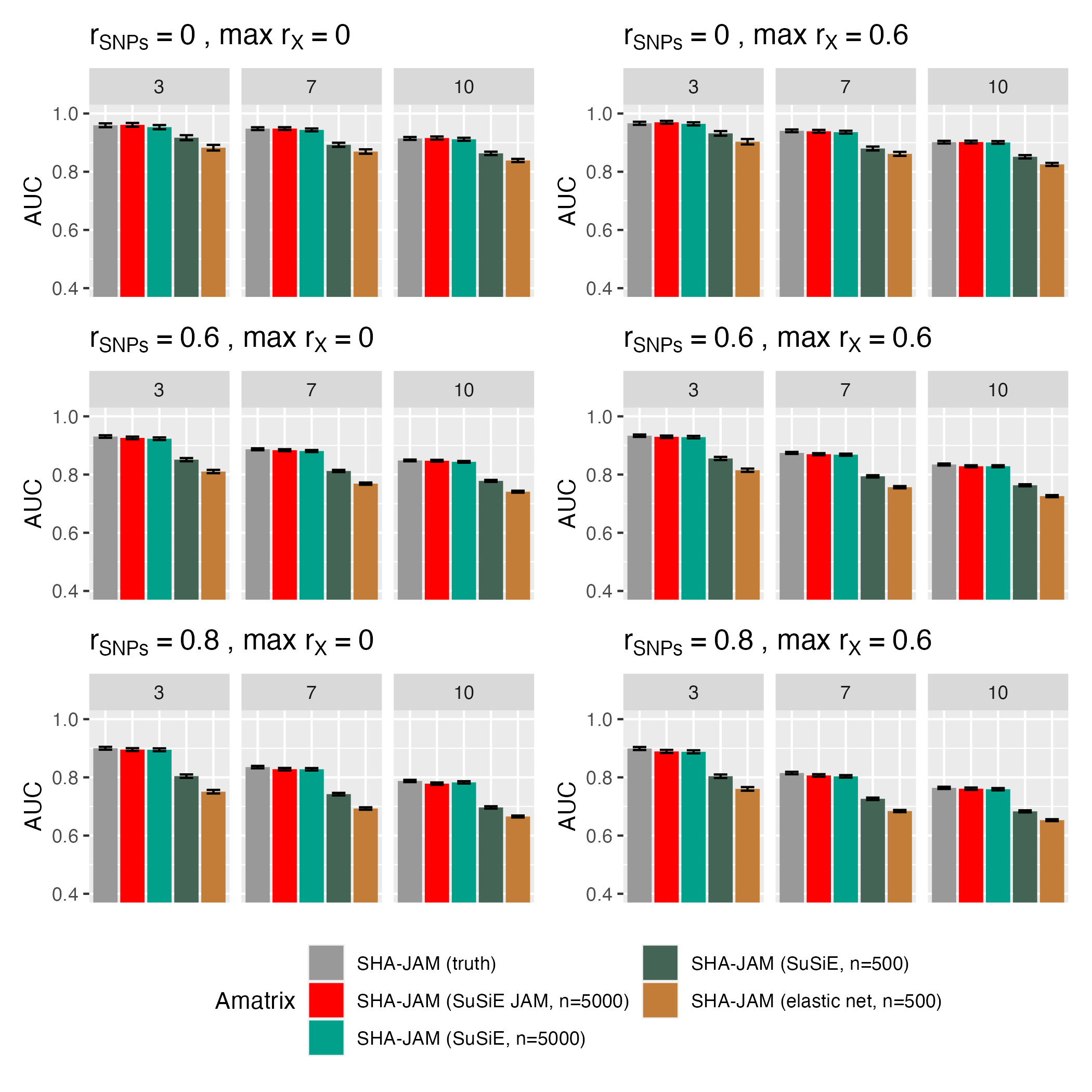


Supplementary Figure 5 Intermediate selection: AUC comparison for SHA-JAM, EN- hJAM and MR-BMA with weight matrices from data of different sample sizes

Simulation results for three LD structures with different number of causal intermediates. The bar plots showed the averaged AUC values and the error bars showed the corresponding standard error. The algorithms are displayed as “Selection algorithm (weight matrix algorithm, n=sample size of the data)”.


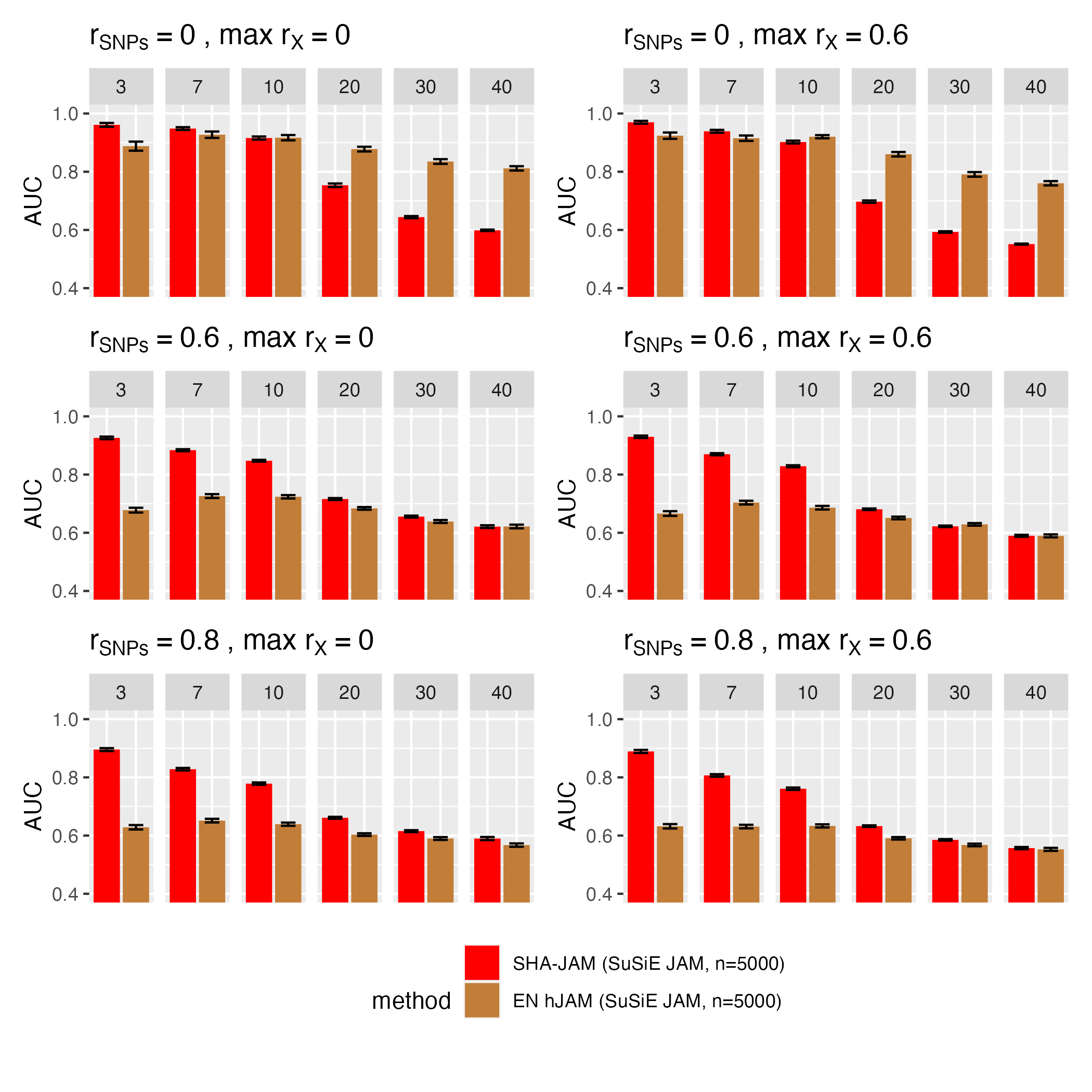


Supplementary Figure 6 Intermediate selection: AUC comparison for SHA-JAM and EN- hJAM with varying number of causal intermediates.

Simulation results for three LD structures with different number of causal intermediates. The bar plots showed the averaged AUC values and the error bars showed the corresponding standard error. The algorithms are displayed as “Selection algorithm (weight matrix algorithm, n=sample size of the data)”.


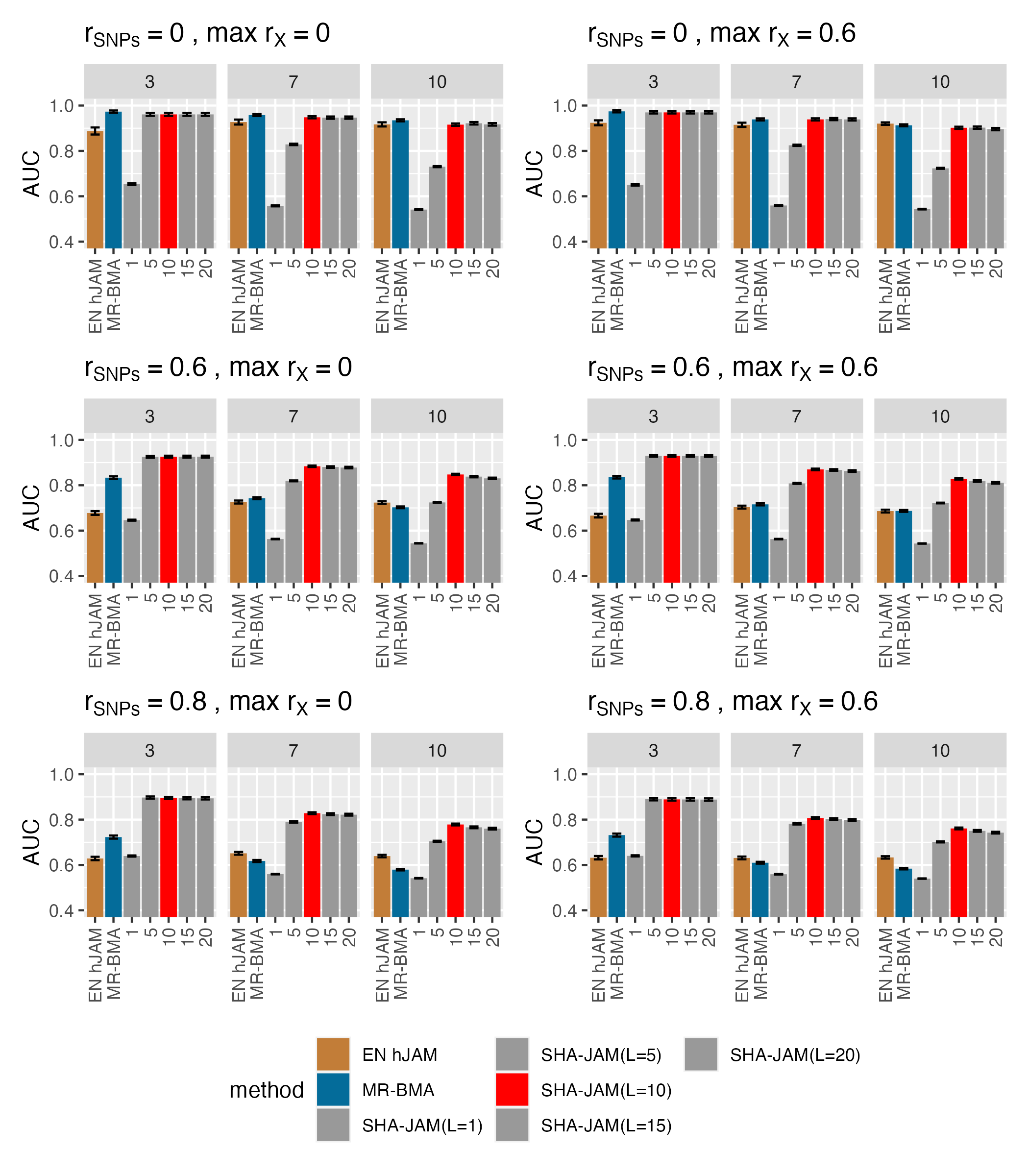


Supplementary Figure 7 Intermediate selection: AUC comparison with varying maximum number of credible sets in SHA-JAM

Simulation results for three LD structures with different number of causal intermediates. Methods include EN-hJAM, MR-BMA and SHA-JAM with maximum number of credible sets (denoted as “L”) varied from 1 to 20. L=10 is used as the default value in other simulation analyses if not specified otherwise. The bar plots showed the averaged AUC values and the error bars showed the corresponding standard error.


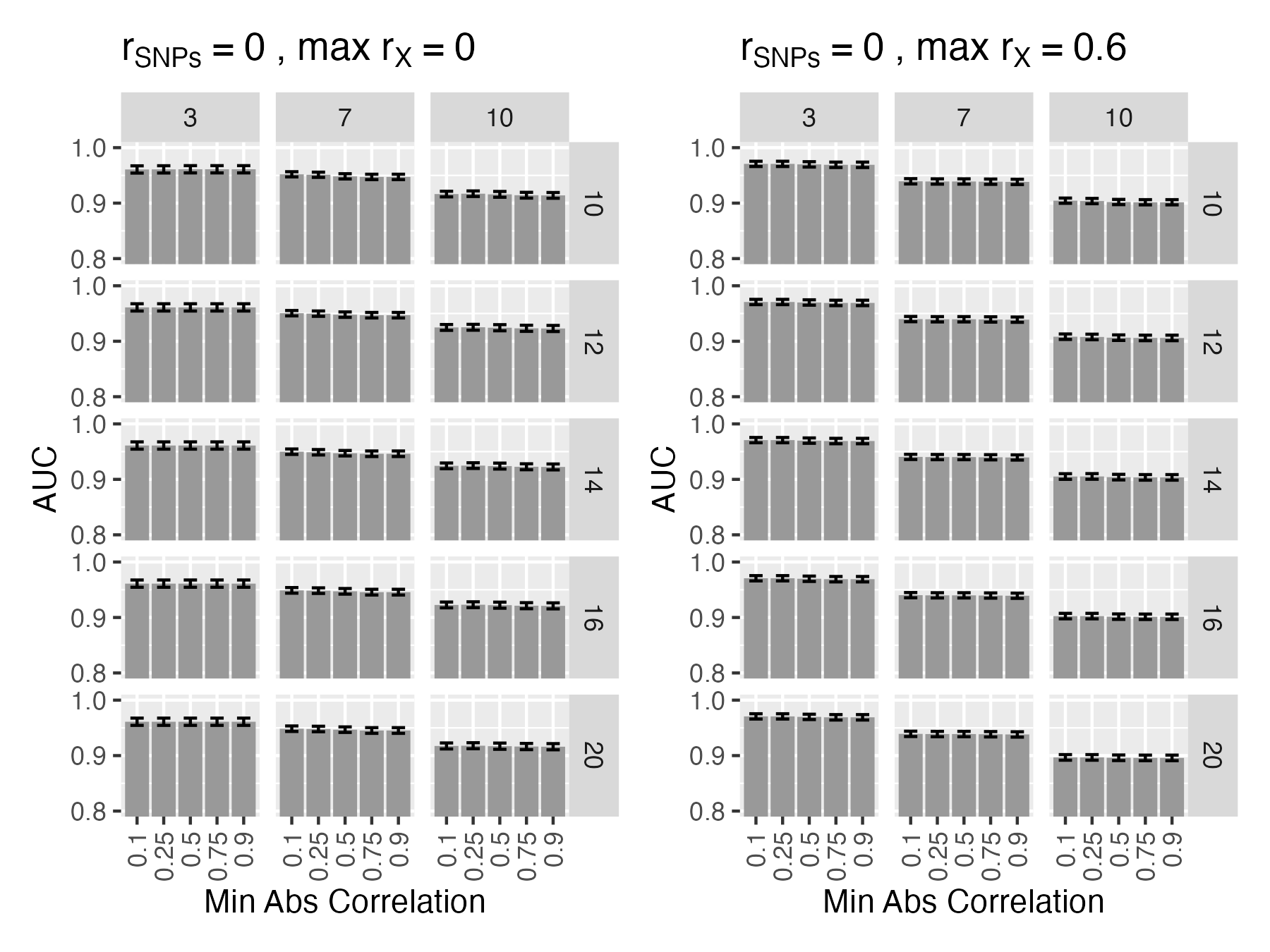


Supplementary Figure 8 Intermediate selection: AUC comparison with varying maximum number of credible sets and varying minimum absolute correlation allowed in a credible set in SHA-JAM

Simulation results for two causal architectures with different number of causal intermediates. Each column in the panel represents the number of true causal intermediates in simulation; each row in the panel represents the maximum number of credible sets (“L”) in SHA-JAM. X-axis shows the different values of minimum absolute correlation in SHA-JAM ("|r|”). The bar plots showed the averaged AUC values and the error bars showed the corresponding standard error.


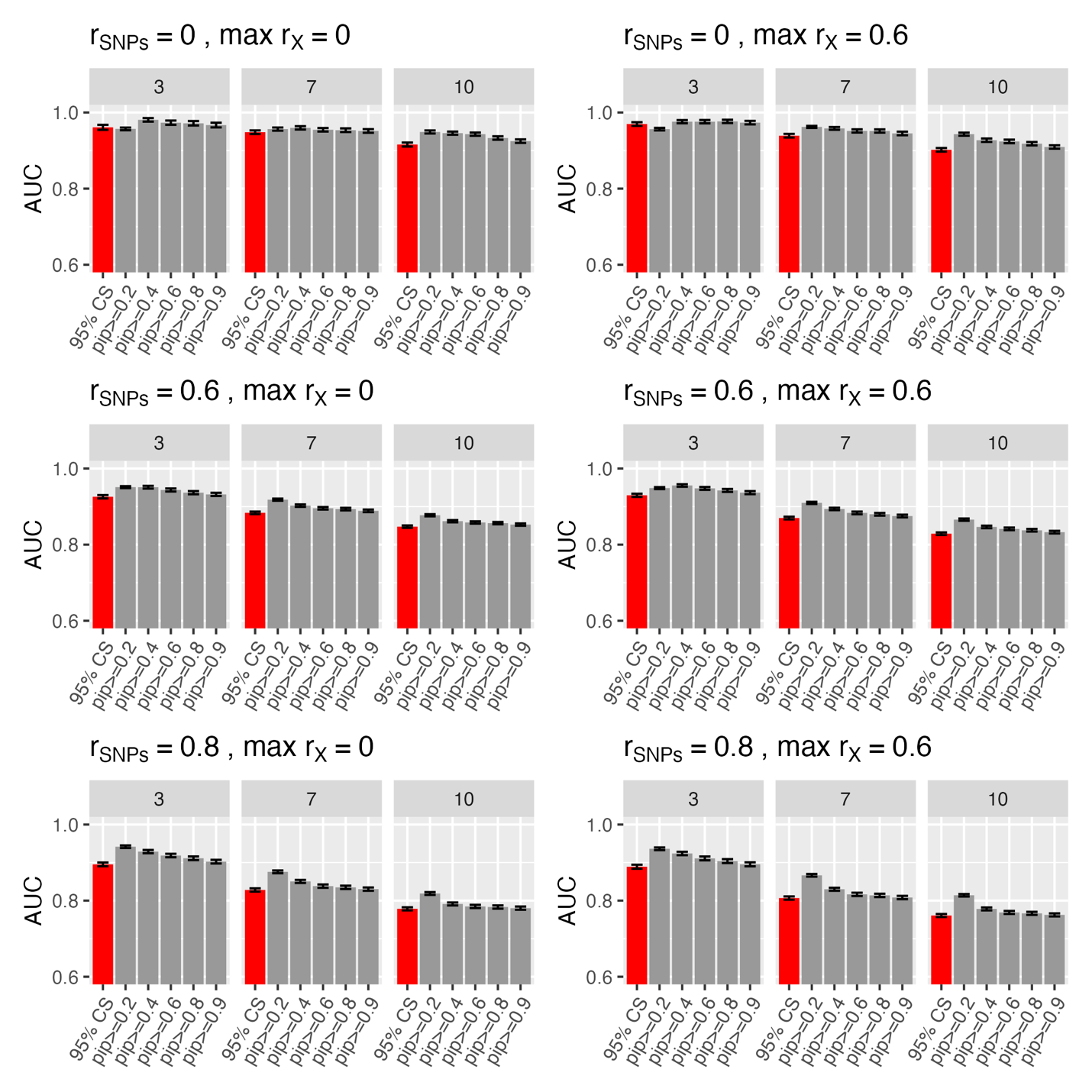


Supplementary Figure 9 Intermediate selection: AUC comparison when intermediates are selected by credible sets (CS) or posterior inclusion probabilities (PIP) in SHA-JAM

Simulation results for three LD structures with different number of causal intermediates. The bar plots showed the averaged AUC values and the error bars showed the corresponding standard error. Results for 95% CS with default parameter settings (maximum number of credible sets = 10 and minimum absolute correlation = 0.5) are shown in red; results for intermediates selected by a hard cutoff of PIP are shown in grey.

**A. 95% Credible Set**

**
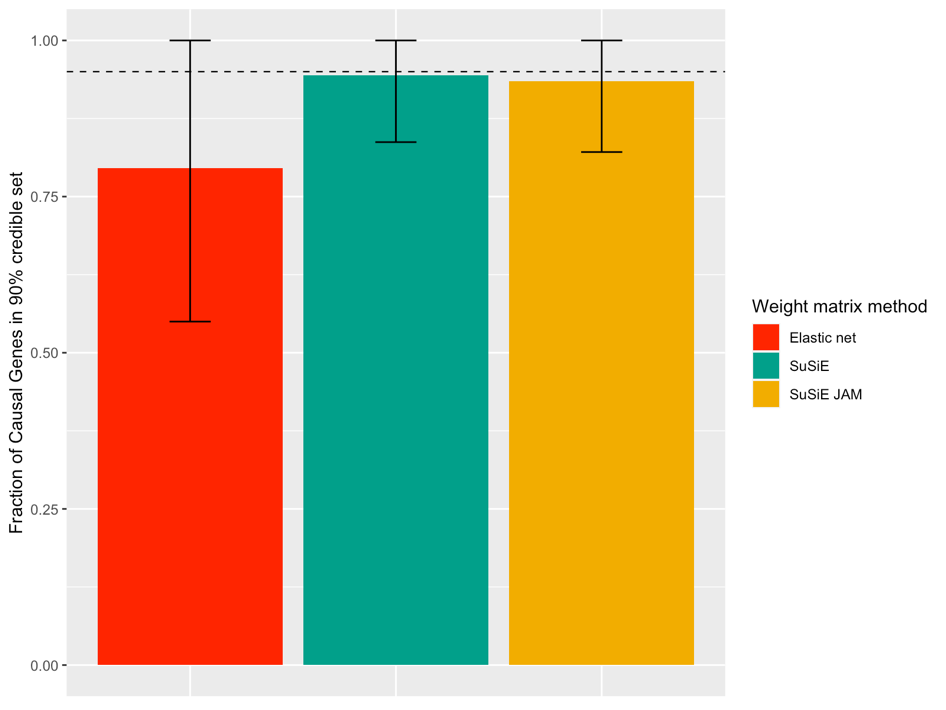
**

**B. 90% Credible Set**

**
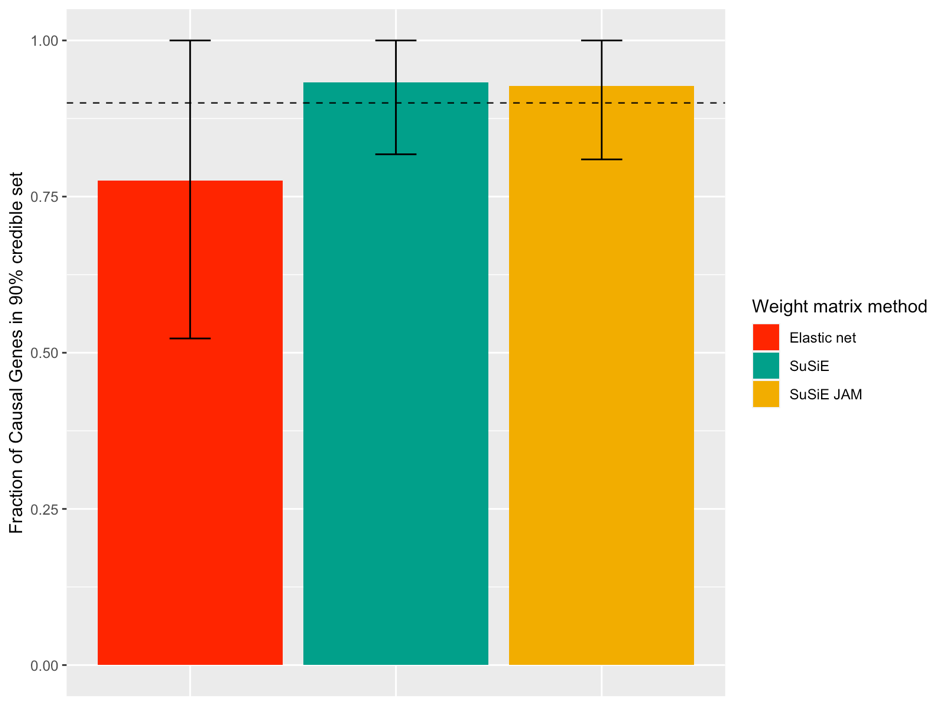
**

**Supplementary Figure 10 Intermediate selection via SHAJAM: Calibration of Credible Sets.** Plots represent the mean and standard error of the proportion of intermediates captured by the 95% (Figure A – dash line) and 90% (Figure B – dash line) credible sets over all simulations with 3, 7 and 10 causal intermediates, a maximum correlation between SNPs of 0.8, and a correlation between intermediates of 0.6. While all approaches use SHAJAM, results are presented for three approaches to construct the weight matrix: elastic net (red); SuSiE using individual person data; and SuSiE using JAM with summary statistics.

| **(a)** | **(b)** |
| --- | --- |
| **** | **** |
| **(c)** | **(d)** |
|  | **** |

Supplementary Figure 11 Diagnosis plots for Cook’s distance and q-statistics

Figure (a) and (b) shows Cook’s distance and q-statistics for IDL.C (K=144) and indicate a influential point, rs2710642, with a large q-statistics of 106. Figure (c) and (d) show Cook’s distance and q-statistics after excluding rs2710642 (K=143) for LDL.D and Est.C+Serum.C. The two figures show an influential point, rs261342, with a Cook’s distance larger than the medium of the F distribution and two other influential points, rs205262 and rs267733, with large q-statistics (q=21.50 and q=21.39, respectively).


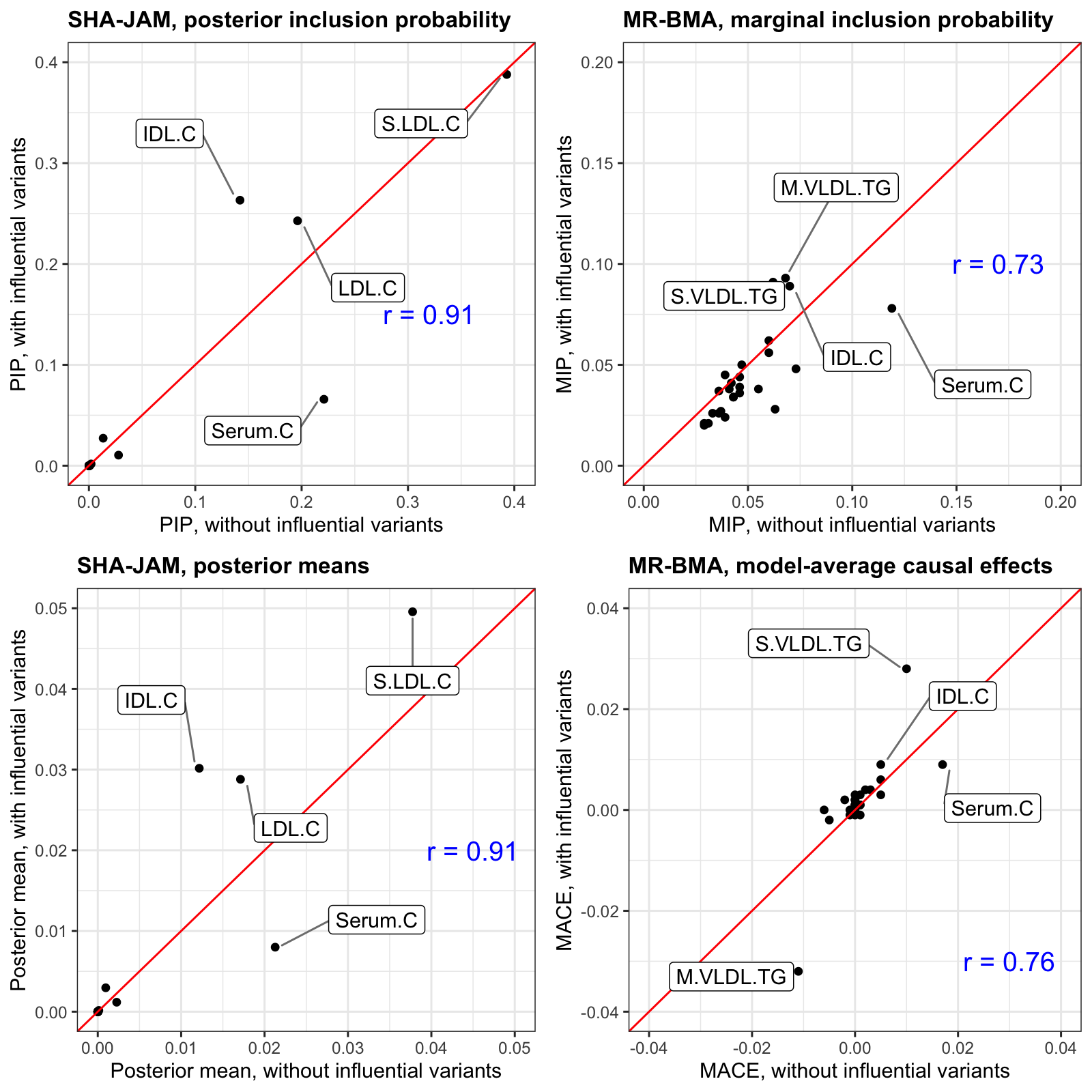


Supplementary Figure 12 Comparisons of the coefficients and inclusion probabilities with and without influential genetic variants for SHA-JAM and MR-BMA.

For SHA-JAM, the coefficients and inclusion probabilities were displayed as posterior means and posterior inclusion probabilities (PIP), respectively. For MR-BMA, the coefficients and inclusion probabilities were displayed as model-average causal effects (MACE) and marginal inclusion probabilities (MIP), respectively. The labeled metabolites were identified by the credible sets of SHA-JAM and the top four ranked metabolites of MR-BMA in the analysis without the influential SNPs, respectively. The Pearson correlation coefficient was applied to show the correlation of parameters of interests with and without the influential variants.

#### References

1. Newcombe, P.J., Conti, D.V., and Richardson, S. (2016). JAM: A Scalable Bayesian Framework for Joint Analysis of Marginal SNP Effects. Genet Epidemiol *40*, 188-201. 10.1002/gepi.21953.

2. Yang, J., Ferreira, T., Morris, A.P., Medland, S.E., Madden, P.A., Heath, A.C., Martin, N.G., Montgomery, G.W., Weedon, M.N., Loos, R.J., et al. (2012). Conditional and joint multiple-SNP analysis of GWAS summary statistics identifies additional variants influencing complex traits. Nat Genet *44*, 369-375, S361-363. 10.1038/ng.2213.

3. Jiang, L., Xu, S., Mancuso, N., Newcombe, P.J., and Conti, D.V. (2021). A Hierarchical Approach Using Marginal Summary Statistics for Multiple Intermediates in a Mendelian Randomization or Transcriptome Analysis. American Journal of Epidemiology. 10.1093/aje/kwaa287.

4. Zou, H., and Hastie, T. (2005). Regularization and variable selection via the elastic net. Journal of the royal statistical society: series B (statistical methodology) *67*, 301-320.

5. Wu, L., Wang, J., Cai, Q., Cavazos, T.B., Emami, N.C., Long, J., Shu, X.-O., Lu, Y., Guo, X., and Bauer, J.A. (2019). Identification of novel susceptibility loci and genes for prostate cancer risk: A transcriptome-wide association study in over 140,000 European descendants. Cancer research *79*, 3192-3204.

6. Mancuso, N., Gayther, S., Gusev, A., Zheng, W., Penney, K.L., Kote-Jarai, Z., Eeles, R., Freedman, M., Haiman, C., and Pasaniuc, B. (2018). Large-scale transcriptome-wide association study identifies new prostate cancer risk regions. Nature communications *9*, 4079.
